# Epithelial transcription factor Elf3 mediates host immune responses to microbiota and protects against aerocystitis in zebrafish

**DOI:** 10.1128/mbio.02267-25

**Published:** 2025-10-28

**Authors:** Briana R. Davis, Colin R. Lickwar, Christiane V. Löhr, Jia Wen, Margaret Morash, Mollie I. Sweeney, Elizabeth L. Reich, Peyton J. Moore, David M. Tobin, John F. Rawls

**Affiliations:** 1 Department of Molecular Genetics and Microbiology, Duke University School of Medicine 12277 , Durham, North Carolina, USA; 2 Oregon Veterinary Diagnostic Laboratory, Carlson College of Veterinary Medicine, Oregon State University 2694, Corvallis, Oregon, USA; 3 Department of Integrative Immunobiology, Duke University School of Medicine 12277 , Durham, North Carolina, USA; Universite de Geneve, Geneva, Switzerland

**Keywords:** host-microbial interaction, transcriptional regulation, symbiosis, zebrafish, aerocystitis, functional genomics, Elf3, germ-free, gnotobiotics, axenic, transcription factor, innate immunity

## Abstract

Animals defend against infections and other diseases by adaptively responding to the microbiota they encounter. These adaptations are driven by changes in gene expression programs; however, our understanding of the transcription factors regulating host responses to microbiota remains limited. By leveraging gene expression and chromatin accessibility data from zebrafish and mice, we identified the epithelial-specific E74-like ETS transcription factor 3 (*Elf3*) as a conserved microbially responsive transcription factor. Transcriptomic analysis of gnotobiotic *elf3* mutant zebrafish larvae revealed that *elf3* is required for a normal host response to microbiota, including induction of immune response genes. Mutation of *elf3* led to immune-related pathologies such as inflammation and infection of the swim bladder, granuloma formation, and reduced survival in adulthood. These results establish *elf3* as an important mediator of host-microbe interactions in zebrafish.

**IMPORTANCE:**

Animals use epithelial barriers to protect themselves from the commensal and pathogenic microorganisms they encounter. These epithelia adapt their function in response to microbial-derived signals, and impairments in these adaptive responses can lead to infection and inflammatory disorders. Improved understanding of the mechanisms underlying host adaptation to microbes can thus be expected to lead to new approaches for promoting health in humans and other animals. Here, we identify the epithelial transcription factor E74-like ETS transcription factor 3 (Elf3) as a mediator of host-microbe interactions in zebrafish. Functional genomic approaches indicated that *Elf3* is upregulated by microbiota in both mouse and zebrafish. Using *elf3* mutant zebrafish, we find that *elf3* mediates induction of host immune responses in larval stages and protects against immune-related pathologies and health deterioration in adults. These results advance our understanding of the transcriptional mechanisms mediating host responses to microbes and provide a new Elf3 deficiency model of epithelial and immune pathology.

## INTRODUCTION

Beginning at birth, animals are exposed to diverse microorganisms that significantly influence their physiology while also posing a constant risk of infection. To manage these complex microbial relationships, animals evolved to deploy epithelial barriers and immune cells at the interfaces between their bodies and the microbial world beyond (1, 2). Homeostasis in these exposed epithelial tissues relies on the capacity of the host cells to adapt their physiology in accordance with the microbial and inflammatory signals they receive. These changes in epithelia and associated immune cell populations are achieved largely through adaptive changes in gene transcription. Identification of mechanisms underlying epithelial and immune cell transcriptional responses to microbiota is therefore an important research goal.

Zebrafish (*Danio rerio*) are useful for investigating mechanisms of host-microbe interactions due to the conservation of their developmental biology and physiology with mammals (3–8), genetic tractability (9), and facile gnotobiotic methods (10, 11). Emerging evidence indicates substantial conservation between zebrafish and mice in their patterns of microbially responsive gene expression and underlying host transcription factors (TFs) within the digestive tract. For example, we previously uncovered conserved microbial suppression of the epithelial TF hepatocyte nuclear factor 4 alpha (Hnf4a) and the epithelial and metabolic gene expression programs it mediates in both zebrafish and mice (12, 13). However, we still have an incomplete understanding of the larger network of TFs used by epithelia and their supporting immune systems to coordinate appropriate responses to microbes.

E74-like ETS transcription factor 3 (Elf3), along with ESE-2 (Elf5) and ESE-3 (Ehf), belongs to a subclass of epithelial-specific (ESE) ETS TFs that have diverse biological roles including epithelial tissue development (14–16), differentiation (17–20), and homeostasis (21). Elf3 is expressed in epithelial-rich tissues in mammals, including the intestine, lung, kidney, and uterus (22–24). Whole animal mutation of *Elf3* in mice leads to variable lethality, with surviving animals showing altered villus architecture in the small intestine and impaired intestinal epithelial cell differentiation (15, 16). In zebrafish, morpholino and clustered regularly interspaced short palindromic repeats (CRISPR) knockdowns of *elf3* have suggested roles in embryogenesis and extracellular matrix (ECM) assembly and organization (25). Gene expression studies in zebrafish indicate that *elf3* is upregulated by microbiota colonization in intestinal enterocytes (26) and goblet cells (27), and is also upregulated in response to pathogenic infections (28–30). In accord, mammalian studies have shown that expression of *Elf3* homologs is responsive to pro-inflammatory stimuli (31–35) and can be mediated by key immunomodulatory TFs like NF-κB in diverse cell types (36–40). While these studies suggest expression of *Elf3* homologs can be induced by microbial and inflammatory signals, the specific roles of Elf3 in host response to microbiota remain untested in any animal system.

## RESULTS

### Identification of Elf3, a candidate TF mediating conserved host responses to the microbiota

**Fig 1:**
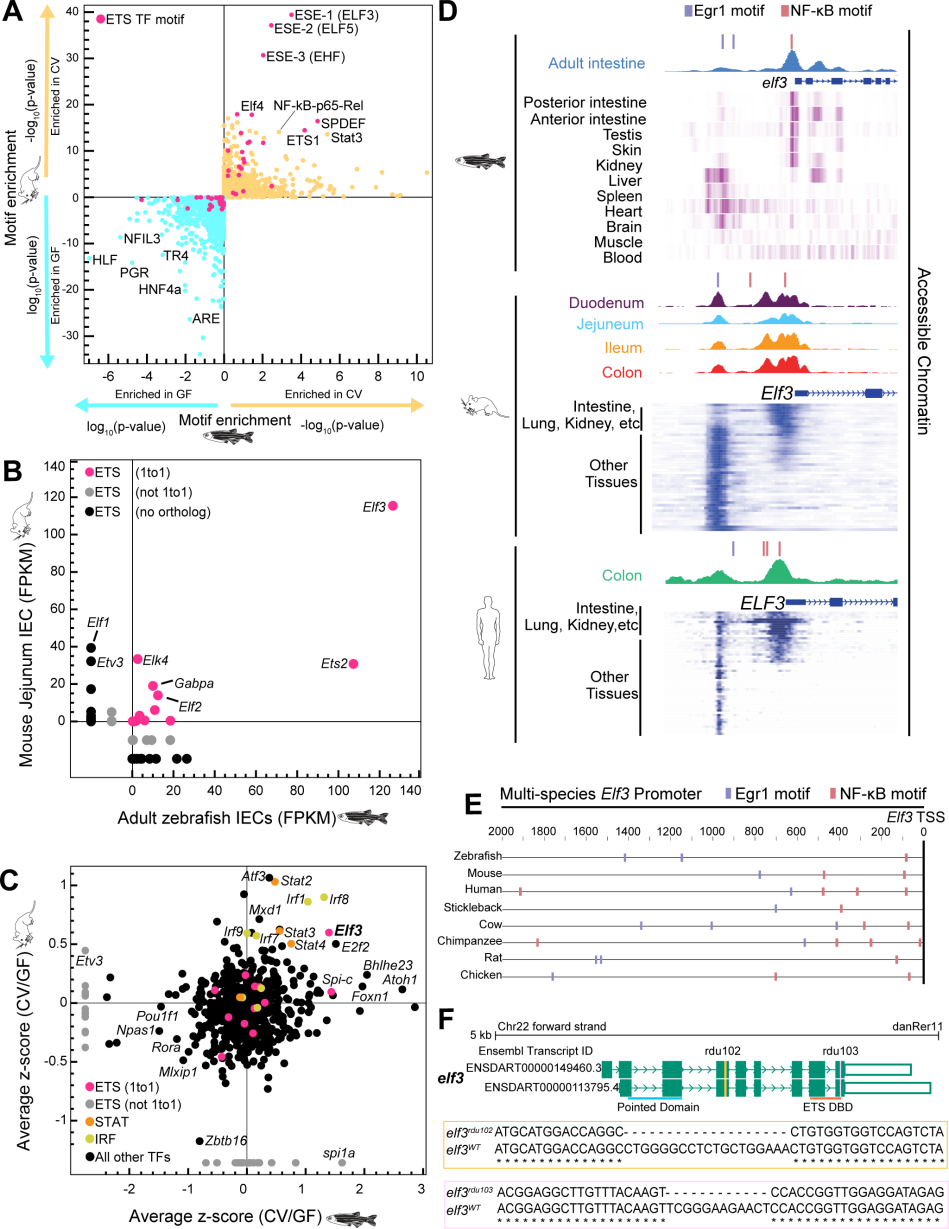
Identification of a candidate TF mediating conserved host responses to the microbiota. (**A**) TFBM enrichment as assessed by default Hypergeometric Optimization of Motif EnRichment (HOMER) software (47) near genes microbially responsive in mouse (*y*-axis) and zebrafish (*x*-axis) digestive tissue. The log_10_(*P*-value) (GF/CV) and −log_10_(*P*-value) (CV/GF) are plotted for motif enrichment. Both comparisons are plotted on each axis for mouse (*y*-axis) and zebrafish (*x*-axis) and are differentiated by the blue and yellow arrows. (**B**) mRNA expression of ETS transcription factors in mouse and zebrafish IECs represented as fragments per kilobase of transcript per million (FPKM) mapped reads. The orthologous relationships between each ETS TF in zebrafish and mice are indicated by the pink (one to one), gray (not one to one), and black (no ortholog) colored data points. (**C**) Average z-scored mRNA levels of genes significantly differential in CV/GF comparisons across multiple mouse and zebrafish studies. (**D**) Visualization of accessible chromatin peaks within the *elf3* promoter of zebrafish (45), mouse (46), and human (45) IECs. Below the accessible chromatin peaks for each species are heatmaps of ATAC-seq performed in diverse zebrafish (51), mouse (52), and human (52) tissue types (visualized via the WashU genome browser [53]). For the mouse and human data sets, more epithelial tissues like the intestine and lung are grouped compared to other tissue types. (**E**) Comparison of the *elf3* promoter across diverse vertebrate species with the location of the conserved Erg1 and NF-κB motifs depicted by the blue and pink boxes, respectively. The vertebrates featured in this analysis include zebrafish (*Danio rerio*), mouse (*Mus musculus*), human (*Homo sapiens*), stickleback (*Gasterosteus aculeatus*), cow (*Bos taurus*), chimpanzee (*Pan troglodytes*), rat (*Rattus norvegicus*), and chicken (*Gallus gallus*). (**F**) Schematic of the protein coding *elf3* isoforms in zebrafish derived from the UCSC genome browser (GRCz11/danRer11). Exons are colored green, and introns are depicted as green arrows. The yellow and pink vertical lines indicate the location of the rdu102 and rdu103 CRISPR lesions, respectively. The horizontal lines at the bottom of the schematic indicate the location of the SMART-predicted functional domains (54, 55), Pointed (blue) and ETS DNA-binding (orange).

We performed a computational screen of intestinal genomic data sets from zebrafish and mouse to identify conserved transcriptional regulatory mechanisms that mediate host responses to microbiota. The intestine was selected because it harbors the largest microbial community in the body (41) and has been the subject of numerous genomic studies of host-microbiota interactions (12, 42–44). We first identified TF binding motif (TFBM) enrichment from intestinal accessible chromatin regulatory regions neighboring genes that were induced or suppressed by colonization of a normal microbiota in germ-free (GF) mice and zebrafish (12, 45–47) (Table S2). We found that in addition to TFBMs for well-characterized inflammatory regulators like NF-κB, interferon regulatory factor (IRF), and signal transducer and activator of transcription (STAT), the regions near genes upregulated by microbiota showed conserved TFBM enrichment for members of the ETS TF family (Fig. 1A). ETS family members can share highly similar motifs, so we further characterized expression levels of ETS family members in intestinal epithelial cells (IECs) from adult zebrafish and mice. We observed that Elf3 was the most highly expressed ETS TF factor in IECs in both animals (Fig. 1B). This is consistent with findings that *elf3* is expressed in diverse epithelia-rich tissues like the intestine and kidney in adult and larval zebrafish (Fig. S1A through D). Next, we leveraged the availability of numerous existing transcriptomic data sets comparing mice and zebrafish reared GF or colonized with microbiota (conventionalized [CV]) to identify TFs that were up- or downregulated by microbiota colonization in the intestine (Table S3). This again revealed that in addition to well-known inflammatory TFs like IRF (48) and STAT (49, 50) family members, Elf3 was consistently induced by microbiota in both species (Fig. 1C). Together, these findings strongly implicate Elf3 as the leading ETS TF candidate and suggest it may be capable of initiating a unique microbial response specifically in epithelial tissues.

We reasoned that conserved tissue-specific expression of *Elf3* and induction by microbiota may be driven by conserved regulatory mechanisms. While DNA sequence conservation levels from teleosts to mammals were low in the *ELF3* proximal promoter, we searched for common modular TFBMs in zebrafish, mouse, and human (45, 56). We only identified proximal NF-κB and more distal Egr1 motifs in common, consistent with known NF-κB sites in the human *ELF3* promoter (38–40) (Fig. 1D). Remarkably, this exact order of motifs relative to the Elf3 transcriptional start site was conserved in a broad range of vertebrate genomes (Fig. 1E). To determine if this motif structure related to chromatin architecture, we queried accessible chromatin from multiple tissues in zebrafish (45, 51), mouse (46, 52), and human (45, 52) and observed two distinct accessible chromatin peaks, each overlapping a different NF-κB or Egr1 motif. Importantly, these peaks frequently showed opposite accessibility across tissues with the NF-κB motif being accessible in epithelial-rich tissues such as intestine and kidney (Fig. 1D). This implies conserved Elf3 function across vertebrate species and suggests a plausible conserved mechanism by which signaling through NF-κB to Elf3 is tissue specific.

### Generation of *elf3* mutant alleles

To test the role of *elf3* in host-microbe interactions and other aspects of zebrafish biology, we used CRISPR-Cas9 to generate zebrafish *elf3* mutant alleles. We isolated two mutant alleles that encode lesions in exons shared by all protein-coding isoforms of *elf3* (Fig. 1F). The *elf3^rdu102^* allele harbors a 19 bp deletion in an early exon that encodes a frameshift mutation and early stop codon. The *elf3^rdu103^* allele harbors a 12 bp deletion near the end of the conserved ETS DNA-binding domain that leads to a four amino acid in-frame deletion, including a highly conserved phenylalanine residue that, when mutated, disrupts ETS domain binding (57, 58) (Fig. S2A). Incrosses between adults heterozygous for either *elf3^rdu102^* or *elf3^rdu103^* yielded larvae at expected Mendelian ratios with no gross abnormalities in morphology or body size, suggesting *elf3* is not required for embryogenesis (Fig. S2B through G). Similar results were obtained using incrossed adults homozygous for either *elf3^rdu102^* or *elf3^rdu103^*, indicating there is no requirement for maternal provision of *elf3* transcript for embryonic development (not shown). We elected to focus our subsequent phenotyping efforts on the in-frame deletion allele *elf3*^rdu103^ to avoid the risk of transcriptional adaptation with other related ETS TFs that might occur in the frameshift *elf3*^rdu102^ allele (59).

### Effects of microbiota colonization and *elf3* genotype on larval gene expression

**Fig 2:**
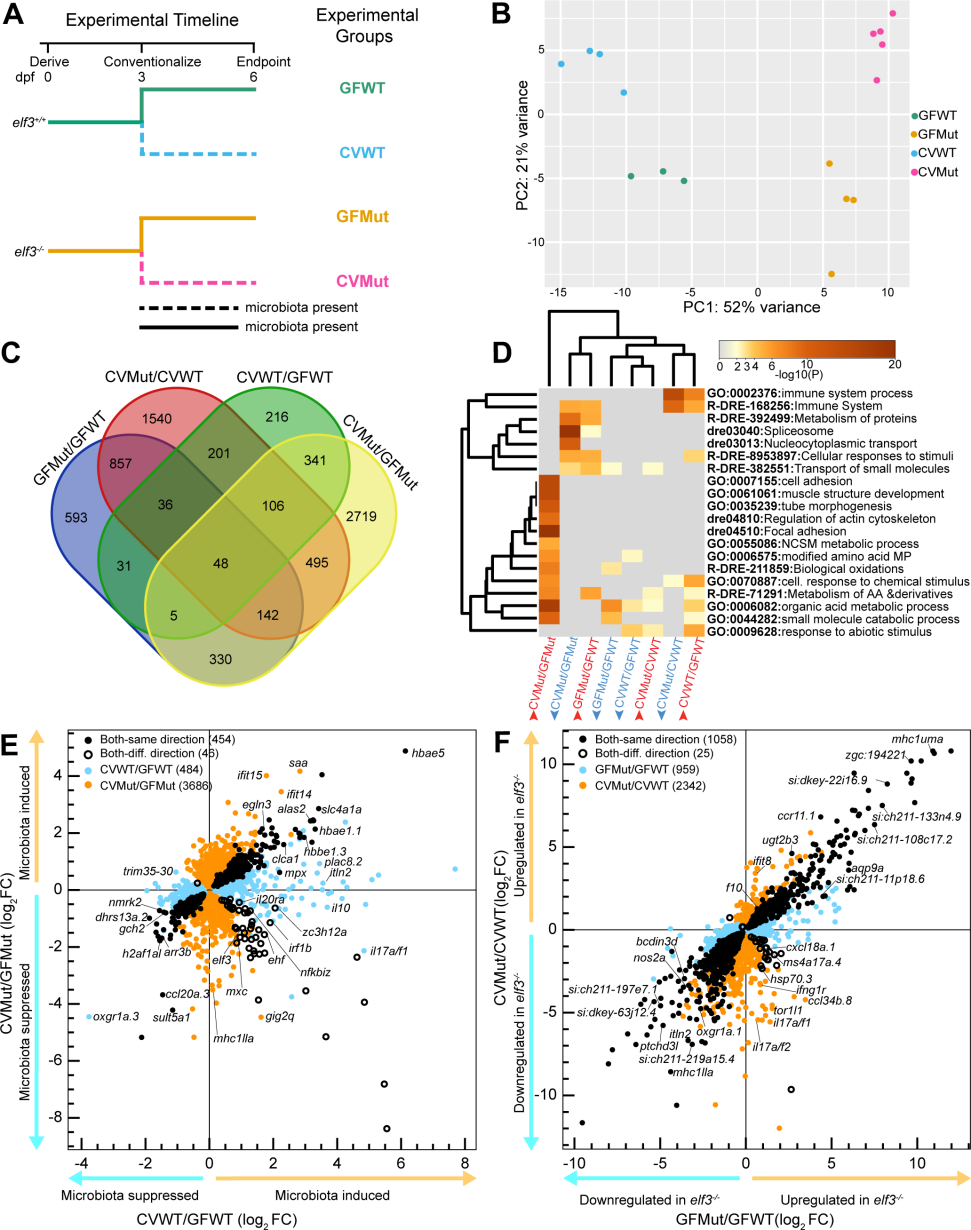
*elf3* genotype and microbial status have significant effects on the larval zebrafish transcriptome. (**A**) Schematic depicting the experimental conditions of the bulk RNA-seq experiment where clutches of *elf3^+/+^* and *elf3^−/−^* larvae produced from homozygous incrosses were derived GF. At 3 days post-fertilization (dpf), half of the larvae from each genotype were colonized with a conventional zebrafish microbiota (CV) (indicated with solid or dashed lines), and all groups were maintained until a 6 dpf endpoint, at which point pooled larval samples were prepared for sequencing (*n* = 6–16 larvae per pool). (**B**) principal component analysis (PCA) plot of the normalized counts for each replicate of the four experimental conditions (GFWT:3, GFMut:4, CVWT:4, CVMut:5). (**C**) Venn diagram overlap of the genes significantly differentially expressed (*P* adjusted < 0.05) in the four comparisons. (**D**) Metascape (61) analysis of the enriched GO terms, biological processes, and signaling pathways that are upregulated (red lettering) and downregulated (blue lettering) in each of the four RNA-seq comparisons. Abbreviations: NSCM metabolic process, nucleobase-containing small molecule metabolic process; modified amino acid MP, modified amino acid metabolic process; metabolism of AAs & derivatives, metabolism of amino acids and derivatives. (**E**) Scatterplot of the gene log_2_ fold change for all significant differentially expressed genes in the CVWT/GFWT and CVMut/GFMut RNA-seq comparisons (*P* adjusted < 0.05), which assess the effect of microbial status on gene expression. (**F**) Scatterplot of the gene log_2_ fold change for all significant differentially expressed genes in the GFMut/GFWT and CVMut/CVWT RNA-seq comparisons (*P* adjusted < 0.05), which assess the effect of *elf3* genotype on gene expression.

**Fig 3:**
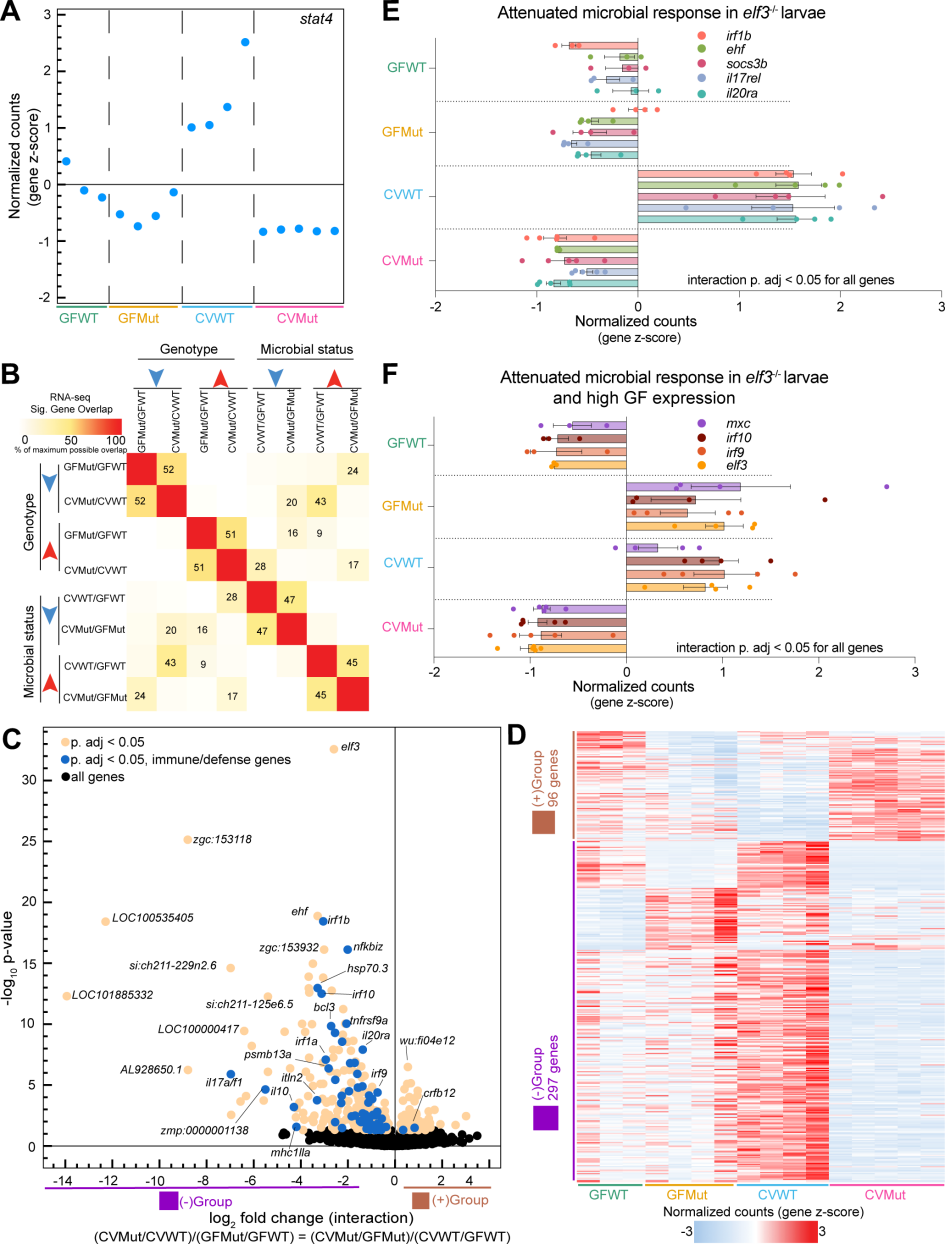
Identification of interaction genes that integrate *elf3* genotype and microbial status. (**A**) Plot of the z-scored normalized counts for significant interaction gene *stat4* where each dot represents one replicate for the indicated experimental condition (*P* adjusted < 0.05). (**B**) Pairwise comparisons of maximum possible overlap between the significant up- or downregulated genes for each of the four RNA-seq comparisons. (**C**) Volcano plot depicting the log_2_ fold change (FC) (*x*-axis) vs −log_10_ *P*-value (*y*-axis) for significant interaction genes identified via likelihood ratio test analysis (*P* adjusted < 0.05). (**D**) Heatmap of the z-scored normalized counts for all significant interaction genes. Out of a total of 393 genes, 96 genes have a positive log_2_FC and 297 genes have a negative log_2_FC (*P* adjusted < 0.05). (**E**) Bar plot of the z-scored normalized counts for genes representative of the attenuated microbial response in *elf3* mutant larvae. All depicted genes are significant interaction genes (*P* adjusted < 0.05). (**F**) Bar plot of the z-scored normalized counts for example immune response genes that fail to be microbially induced in *elf3* mutant larvae at a magnitude comparable to wild-type and that additionally exhibit high expression in GF conditions. All depicted genes are significant interaction genes (*P* adjusted < 0.05).

**Fig 4:**
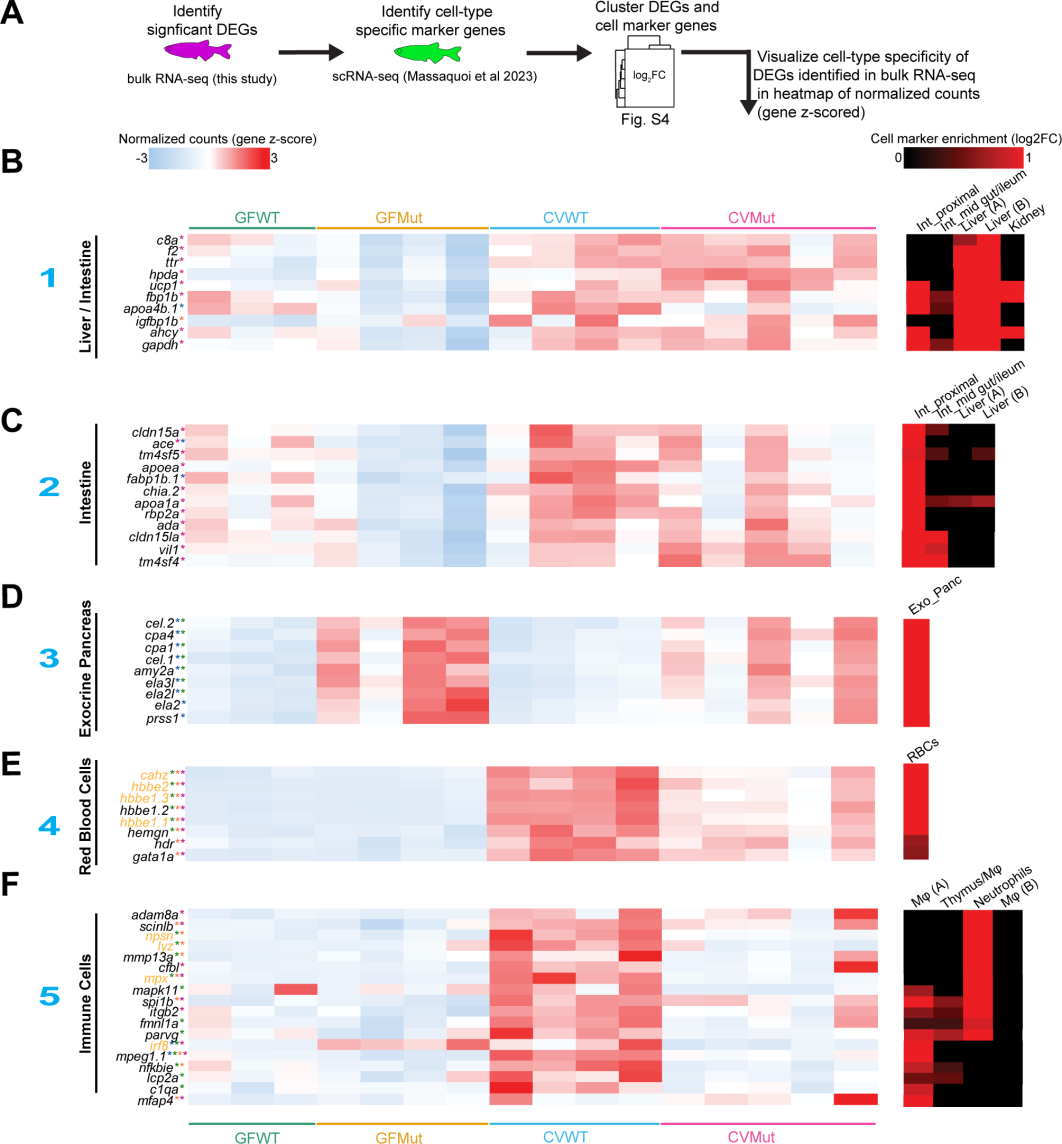
Differentially expressed genes in larval RNA-seq exhibit cell-type specificity. (**A**) Schematic of application of cell type-specific calls to significant differentially expressed genes (DEGs) in the RNA-seq data set. Significant DEGs were identified and clustered with cell marker genes identified in an scRNA-seq data set generated from the disassociated cells of 6 dpf zebrafish (27). The resulting clustered heatmap is featured in Fig. S4, and the heatmaps below are visualizations of example genes for each cell type. (**B**) Heatmaps of the gene z-scored normalized counts for diverse cell markers such as the liver/intestine (**B**), intestine (**C**), exocrine pancreas (**D**), red blood cells (RBCs) (**E**), and immune cells (**F**). All genes are significant in at least one of the four RNA-seq comparisons (*P* adjusted < 0.05). The color of the asterisk next to each gene indicates in which comparison(s) the gene is significantly differential (CVMut/GFMut: maroon, CVWT/GFWT: orange, GFMut/GFWT: blue, and CVMut/CVWT: green). Genes in yellow font are significant interaction genes. The blue numbers to the left of each heatmap indicate the cell type cluster the example marker genes represent (Fig. S4). The heatmaps on the far right show the log_2_ fold change of the indicated gene as a function of cell marker enrichment (27). Abbreviations: int_proximal, intestine_small/proximal; int_mid gut/ileum, intestine mid gut/ileum; exo_panc, exocrine pancreas; Mφ, macrophages.

To test *elf3*’s roles in larval zebrafish and host-microbiota interactions, we performed bulk RNA sequencing on GF and CV wild-type (WT) and mutant (Mut) larvae at 6 days post-fertilization (dpf) (Fig. 2A). Principal component analysis of the normalized RNA-seq counts revealed that biological replicates for the four conditions stratified based on microbial status and genotype (Fig. 2B). This stratification was consistent with the identification of hundreds of significant differentially expressed genes in four comparisons (i.e., microbial effect in CVWT/GFWT and CVMut/GFMut, and genotype effect in GFMut/GFWT and CVMut/CVWT) (Fig. 2C; Table S4). We observed no clear differential expression of developmental markers between WT and mutant larvae (Fig. S3; Table S5), which was consistent with our larval morphometric analyses (Fig. S2), together indicating that the differential gene expression we observed here is not caused by differences in developmental progression (60). We continued our RNA-seq analyses to highlight four separate representations: (i) the effect of *elf3* genotype on transcriptional responses to microbiota (Fig. 2E), (ii) the effect of *elf3* mutation on host gene expression independent of microbial status (Fig. 2F), (iii) the interaction between genotype and microbial status (Fig. 3), and (iv) the underlying tissue specificity of the differentially expressed genes (Fig. 4)

### *elf3* mediates host responses to the microbiota in larval zebrafish

To understand if *elf3* mutation affects transcriptional responses to microbiota, we first defined the normal host response as those genes differentially expressed in the CVWT/GFWT comparison (Fig. 2E; Table S4). Functional enrichment analysis (61) indicated that microbial colonization induced expression of immune and defense response genes like *itln2*, *npsn*, and *mpx,* similar to previous larval bulk transcriptomic studies (62) (Fig. 2D; Table S6). In agreement with our analyses in Fig. 1, we also observed upregulation of *elf3* upon colonization (Fig. 2E). Consistent with previous studies (12), the microbiota suppressed genes involved in abiotic responses like circadian rhythm (*cry2*, *per1a*, *bhlhe41*) as well as organic acid metabolic processes such as fatty acid metabolism (*cpt1b*, *hadhaa*, *angptl4*) (Fig. 2D; Table S6). Together, these data identify genes differentially expressed in response to microbiota in WT larvae.

Next, we assessed how the response to microbiota is affected by *elf3* mutation. We found 46% of the differentially expressed genes in the CVWT/GFWT comparison were similarly differential in the CVMut/GFMut comparison, indicating that there are substantial aspects of the host response to microbiota that are shared between genotypes (closed black circles in Fig. 2E). Enrichment analysis of this shared transcriptional program suggested that *elf3* is not required for microbial upregulation of many processes, such as gas transport and hypoxia response, or suppression of iron homeostasis and phototransduction (Table S6).

Beyond these similarities, we observed extensive differences in the transcriptional response to microbiota in WT and *elf3* mutant larvae (Fig. 2D and E). Because *elf3* is frequently upregulated in response to microbiota (Fig. 1), we were particularly interested in differences between the genes upregulated in the CVWT/GFWT comparison and downregulated in the CVMut/CVWT comparison (Fig. 2E; Table S4). We were intrigued to find that genes involved in immune response were among those that differed in those two comparisons. For example, the immune system process was the top enriched biological process for genes induced by microbiota in WT animals, but it was not among the top 20 enriched terms in the *elf3* mutant response to microbiota (Fig. 2D; Table S6). In fact, the same immune system process category was the top enriched process downregulated in the CVMut/CVWT comparison, suggesting that *elf3* mutants fail to mount a normal immune response to microbiota (Fig. 2D; Table S6). These *elf3*-dependent immune response genes included those involved in cytokine receptor signaling (63, 64) (*il10, il22*, and *tnfsf10*) and immune cell differentiation (65, 66) (*ikzf1*, *cebp1*) (Fig. 2E; Table S4). Furthermore, we were interested to find that a small subset of genes that were induced by microbiota in WT but suppressed by microbiota in *elf3* mutants were enriched for immune system functions and C-type lectin receptor signaling (Table S6; open black circles in Fig. 2E). This set included IRF TFs (*irf1a, irf1b, irf9, irf10*) (Table S4). Together, these data suggest that microbial induction of immune genes observed in WT zebrafish is attenuated in *elf3* mutants.

We next evaluated how *elf3* mutation impacts the pattern of genes downregulated by microbiota. Genes downregulated in response to microbiota in WT were enriched for organic acid metabolism and abiotic responses, which included circadian rhythm regulators (*cry2*, *per1a, cipca*) (67). However, those same pathways were not similarly suppressed in mutants (Fig. 2D; Table S6). These data highlight a putative role for *elf3* in mediating microbial influences on circadian regulation.

### *elf3* regulates gene expression in larval zebrafish independent of microbial status

Having evaluated the impact of *elf3* mutation on host response to microbiota, we next wanted to evaluate how *elf3* mutation affects gene expression beyond the microbial response. To do this, we identified *elf3*-dependent genes that are similarly differential in both the CVMut/CVWT and GFMut/GFWT comparisons. We found that 52% of *elf3*-dependent genes in the GF condition were similarly up- or downregulated in both microbial conditions (Fig. 2D, closed black circles in Fig. 2F). The shared upregulated program was enriched with genes involved in mRNA stability, proline metabolism, and response to cellular stress (Table S6). Conversely, pyruvate and glycerophospholipid metabolic pathways (Table S6), along with mucins and mucin-associated genes (*muc13a, muc5e, mucms1*) (Table S4), were downregulated independent of microbial status in *elf3* mutants. We also identified defense response genes (30, 68) (*irg1l, nos2a, crp7*) among the shared downregulated genes, indicating potential immunologic roles for *elf3* independent of microbial stimulation (Table S6). Together, these results uncover diverse *elf3*-dependent transcriptional programs in zebrafish larvae.

### Interaction genes integrate microbial status and *elf3* genotype

After examining the separate effects of microbial status and *elf3* genotype on the larval transcriptome, we investigated the effects of interactions between both variables on gene expression. For example, transcription factor *stat4* is downregulated in mutants and fails to be microbially induced at a magnitude comparable to WT (Fig. 3A), suggesting its expression results from the integration of both genotypic and microbial information. When we compared the overlap of up- and downregulated genes for each of our four RNA-seq comparisons, we noted strong overlap (43%) between genes upregulated in the CVWT/GFWT comparison and those downregulated in the CVMut/GFMut comparison (Fig. 3B). This overlap, which is representative of genes like *stat4*, was consistent with our functional enrichment analysis (Fig. 2D). In contrast, there was less (28%) overlap between genes downregulated in the CVWT/GFWT comparison and those upregulated in CVMut/CVWT (Fig. 3B). These data support the predictions of our functional genomic meta-analyses (Fig. 1) that *elf3* is preferentially required for microbially induced responses.

As a more stringent approach to interrogate interactions between *elf3* genotype and microbial status, we performed likelihood ratio test (LRT) analysis on our RNA-seq data (13, 69). This LRT analysis identified several gene expression patterns that have either a negative or positive interaction log_2_ fold change that describes how the genes behave across our four conditions (Fig. 3C and D). Immune processes like cytokine signaling and major histocompatability complex (MHC) class I antigen presentation were enriched in the negative log_2_ fold change interaction gene signature (Table S6). In fact, approximately 20% of this interaction signature can be classified as putative immune response genes (Fig. 3C; Table S6). A closer inspection of immune gene expression across all four conditions revealed three major patterns. The first pattern consisted of immune genes that are induced by microbiota in WT but not *elf3* mutants like *stat4* and other immune-responsive transcription factors (*irf1a, irf1b*) as well as mediators of cytokine signaling (*il17rel, il20ra, soc3b)* (Fig. 3E). Notably, we also identified ETS TF *ehf,* which has emerging roles in inflammation and immune response (40, 70, 71), in this pattern (Fig. 3F). The second pattern identified immune genes that failed to be microbially induced in *elf3* mutants but exhibited high expression in GF mutants. The e*lf3* gene fell into this category, suggesting that *elf3* might regulate its own expression in addition to expression of immune regulators like IRF TFs (*irf9*, *irf10*) and genes important for viral responses (*mxc*) (Fig. 3F). Lastly, we also observed immune genes (72, 73) like *sting1, ifi35,* and *malt3* that were significantly downregulated in mutants in CV conditions despite not being significantly upregulated by microbiota in WT larvae (Table S4). Together, these data indicate that *elf3* mutants have an attenuated immune response to microbial colonization. Interestingly, the positive log_2_ fold change interaction (96 out of total 393 genes) signature revealed upregulation of intermediate filament and extracellular matrix components specifically in mutants under CV conditions, suggesting that these components might be compensatory for the blunted immune response (Table S6).

### Mapping differentially expressed genes in larval bulk-RNA seq to cell-type specificity

Since the bulk RNA-seq data captured responses throughout the whole larvae, we sought to identify the cellular origins of the gene expression differences. Thus, we performed hierarchical clustering of all differentially expressed genes in the RNA-seq data and previously annotated cell-specific marker genes (Fig. 4A) (27). This revealed that many genes differentially expressed as a function of microbial status and *elf3* genotype are also strongly expressed by distinct cell types (Fig. S4A through E). For example, *elf3* genotype had significant effects on the expression of pancreatic marker genes (Fig. 4D). We found pancreatic markers like *cel.1*, *cel.2*, and *cpa1* were upregulated in mutants in both microbial conditions (Fig. 4D). This is interesting since a previous study reported that Elf3 expression levels increase during pancreatic development from embryonic days E12.5 to E18.5 in mice (74), and *elf3* mediates epithelial identity genes in a pancreatic adenocarcinoma cell line (75). Microbe-independent roles for Elf3 in epithelial tissue differentiation are also supported by the literature (15, 16, 76).

We also found microbially responsive transcriptional programs associated with specific tissues only in the *elf3* mutant genotype. For example, several genes were specifically downregulated in mutants in GF conditions that were also marker genes for digestive tissues like the intestine (*apoea*, *cldn15a*, *vil1*) and liver (*f2*, *c8a*) (Fig. 4B and C). Conversely, neuronal marker genes like *eno2, nrsn1,* and *sox4a* were upregulated in mutants in GF conditions (Fig. S4E).

Finally, we looked for cell-specific marker genes that followed the same gene expression pattern as the attenuated immune response we observed in colonized *elf3* mutants. Red blood cell (RBC) and immune cell marker genes followed this pattern (Fig. 4E and F). We observed blunted microbial induction of RBC (*hbbe1.1*, *hbbe1.3, hbbe2*) and leukocyte (*lcp1, coro1a, ikzf1*) marker genes (Table S4). We also found that several highly specific neutrophil marker genes (*lyz, npsn, mpx, mmp13a*) were microbially induced in WT but not mutant larvae (Fig. 4F). These data, taken together, suggest that *elf3* function regulates specific aspects of the hematopoietic response to colonization.

### *elf3* mutation reduces adult zebrafish survival

**Fig 5:**
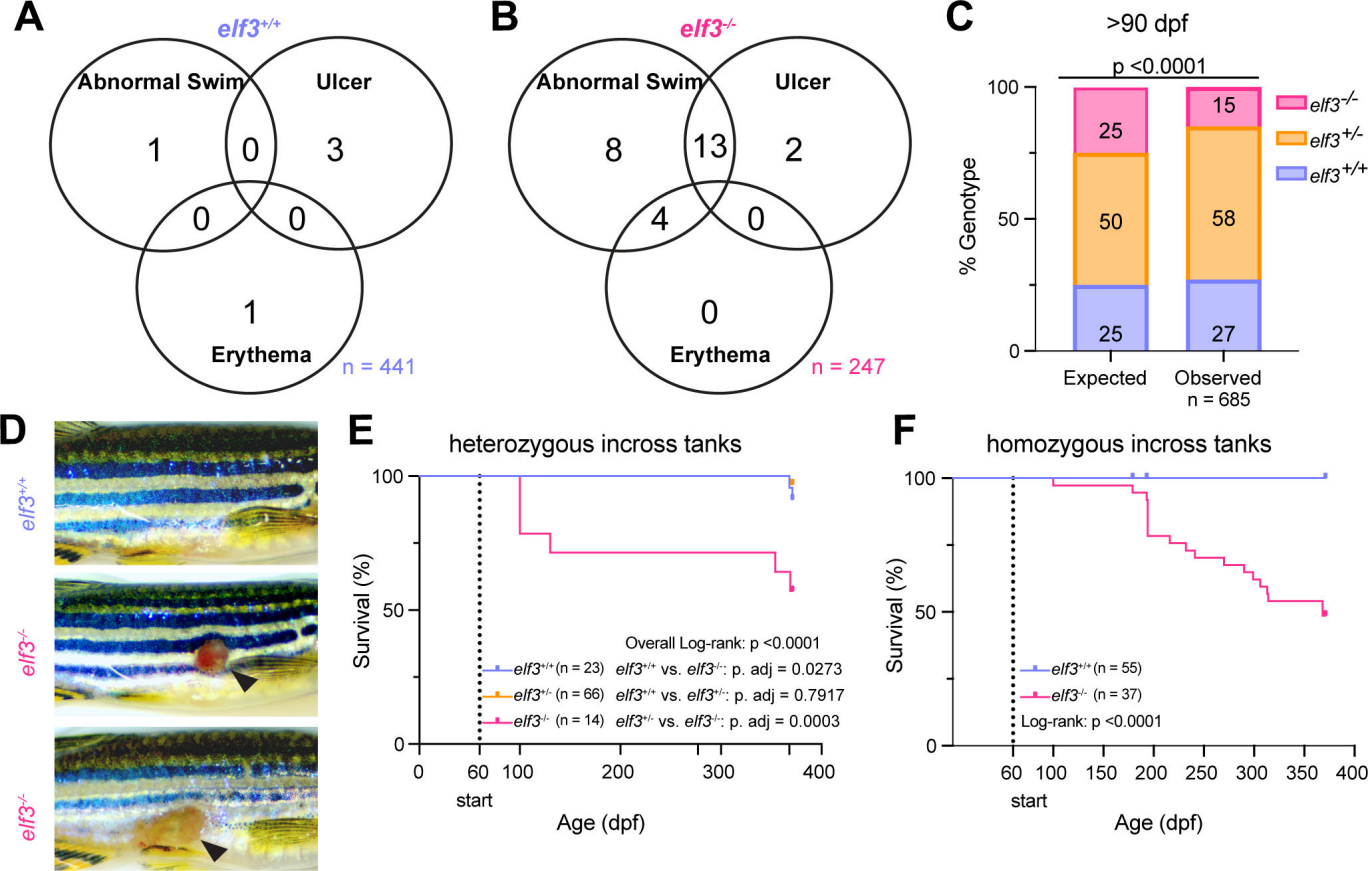
*elf3^−/−^* adults present immune-related pathologies and exhibit poor survival. (**A, B**) Venn diagram of observed clinical signs of health deterioration, such as abnormal swimming, erythema, and ulcer development in *elf3^+/+^* (**A**) and *elf3^−/−^* (**B**) adults. (**C**) Comparison of the genotypic ratios observed in adult zebrafish >90 dpf generated from heterozygous incrosses of *elf3*^+/−^ parents to expected Mendelian outcomes. (**D**) Representative images of severe (bottom) and milder (middle) ulcers in *elf3^−/−^* adults compared to healthy *elf3^+/+^* adults (top). (**E**) Kaplan-Meier survival curves of co-housed *elf3* genotypes starting from the initial observation at 60 dpf (dotted line) and ending at 371 dpf. Repeated genotyping events occurred at 60 dpf, 100 dpf, 277 dpf, and 368 dpf. (**F**) Kaplan-Meier survival curves of separately housed *elf3^+/+^* and *elf3^−/−^* adults starting from the initial observation at 60 dpf (dotted line) and ending at 371 dpf. *P*-values were calculated for panel **A** using a chi-square goodness-of-fit test and for panels **E and F** using an overall log-rank test followed by Bonferroni-corrected log-rank tests for individual comparisons when required.

Since *elf3* mutants fail to induce an appropriate immune response to the microbiota, we hypothesized that *elf3* mutant fish might develop immune-related pathologies. Indeed, adult *elf3* mutants of both sexes presented a range of clinical pathologies such as abnormal swimming behavior indicative of impaired swim bladder buoyancy, ulcer development on the body flank, or erythema (Fig. 5A and B). Approximately 11% of evaluated mutants (27 out of 247) presented one or more of these observable pathologies compared to 1% of observed WT adults (5 out of 441) (Fig. 5A and B). Overall, *elf3* mutant adults exhibit poor survival fitness, as we recovered ≥40% fewer mutants than expected when genotyping both alleles at adulthood (Fig. 5C; Fig. S5A and B).

To track the reduced survival, we longitudinally assayed animals in mixed *elf3* genotype heterozygous tanks as well as separate homozygous WT and mutant tanks starting from 2 months to approximately 12 months of age (Fig. 5E and F; Fig. S5C through E). Repeated genotyping of fish from heterozygous tanks revealed a 43% reduction in mutant survival by 12 months of age (Fig. 5E). To rule out that the *elf3* mutant survival effect was driven by competition between genotypes, we assessed survival in homozygous tanks and observed similarly reduced survival (51%) (Fig. 5F).

### *elf3* moribund adults exhibit swim bladder inflammation

**Fig 6:**
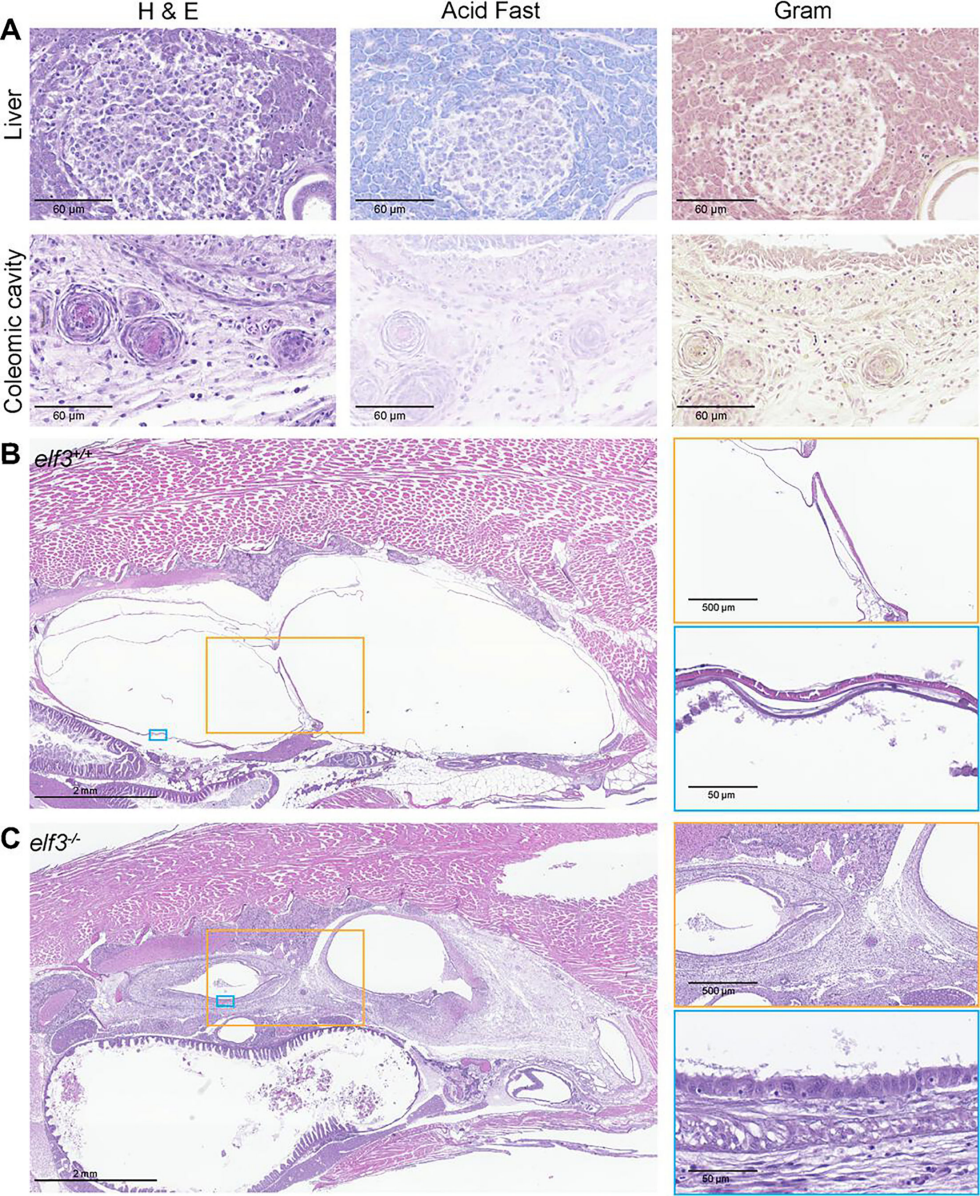
Moribund *elf3^−/−^* adults develop aerocystitis. (**A**) Representative images of granulomas that can develop in moribund *elf3^−/−^* adults in the liver and coelomic cavity. For each example granuloma, there is a hematoxylin and eosin (H&E)-stained image, an acid-fast-stained image, and a Gram-stained image to assess the overall morphology of the granuloma as well as to test for the presence of *Mycobacterium*. (**B, C**) Representative images of the coelomic cavity of healthy appearing, wild-type controls (**B**) and moribund mutant (**C**) adults (histopathological evaluation of 12 moribund mutants and 12 wild-type controls). The two inset images featured below are magnified images of the swim bladder that highlight the inflammation observed in the anterior and posterior chambers of the moribund *elf3* mutant. Scanned images were rotated or vertically reflected for consistent body orientation across all images.

To understand the pathologies associated with reduced *elf3* mutant survival, we performed histological analysis on 12 moribund *elf3* mutants and healthy wild-type controls. Adults were defined as moribund based on presentation of the aforementioned clinical signs (abnormal swimming, ulcer, and/or erythema). Four moribund mutants had granulomas in locations including the liver, ovary, and coelomic cavity, while no granulomas were detected in WT animals (Fig. 6A). Mycobacterial infections are a common cause of granulomas in zebrafish (77–79), so we used detection methods such as acid-fast stain (80) as well as an infection assay (81) to assess susceptibility. All the granulomas were acid-fast negative, suggesting non-mycobacterial contributions to granuloma formation (Fig. 6A). When we injected standard doses of *Mycobacterium marinum* into 2 dpf wild-type and *elf3* mutant larvae, we observed no significant difference in bacterial burden between genotypes at 5 days post-infection (or 7 dpf) (Fig. S6A). Therefore, while *elf3* appears to be required for aspects of the immune response to microbiota in larvae (Fig. 2), it does not alter susceptibility to *M. marinum* infection during larval stages.

Our histological analysis also revealed extensive inflammation of the swim bladder and surrounding tissue (i.e. aerocystitis and peri-aerocystitis) in 11 out of 12 *elf3*^−/−^ adults, whereas 0 out of 12 *elf3^+/+^* controls had minimal inflammation between swim bladder compartments (Fig. 6B and C). To test if the aerocystitis is associated with bacterial infection, the swim bladders of moribund *elf3* mutant zebrafish displaying aberrant swimming behavior and WT controls were removed, homogenized, and plated on rich media to evaluate the bacterial load. Whereas none of the tested WT animals (*n* = 4) showed significant bacterial burden in the swim bladder, the swim bladders in four out of five of the *elf3* mutant animals yielded abundant bacterial growth with a single colony morphology emerging from each animal. 16S rRNA gene sequencing of the isolated bacteria revealed that two mutants were infected with hemolytic *Vibrio cholerae* and two mutants were infected with nonhemolytic *Pseudomonas oleovorans*. Together, these results indicate that moribund status in adult *elf3* mutants is associated with aerocystitis, peri-aerocystitis, accompanying swim bladder infection, and/or non-mycobacterial granuloma formation.

We were curious if these histopathological phenotypes arose prior to reaching moribund status. Histopathological examination of *elf3* mutant and wild-type fish approximately 5 months in age revealed that *elf3* mutation does not result in significant clinical presentations in apparently healthy adults (data not shown). These data combined with our survival and clinical observations support a working model wherein *elf3* mutant adults are more susceptible to sporadic infection-associated aerocystitis, peri-aerocystitis, and granuloma formation, leading to abnormal swimming behavior and ultimately death.

## DISCUSSION

Our findings here establish Elf3 as a microbially regulated TF that is also required for mediating diverse host responses to microbiota. Our observation that *Elf3* genes are commonly induced by commensal microbiota in digestive tissues in zebrafish and mice is in accord with previous studies showing *Elf3* homologs are induced by bacterial pathogens (28–30) and symbionts (26, 27, 82) in diverse animal species. Previous literature indicated many roles for *elf3,* including regulation of epithelial identity and differentiation (15, 16, 76, 83), inflammation (31–40), ECM organization (25), and epithelial-mesenchymal transition (83–93). However, it remained unclear if *elf3* could integrate information derived from microbes to affect downstream host transcriptional processes and whether this would uncover additional physiologic roles. By comparing the transcriptomes of mutant and wild-type zebrafish larvae reared in GF and CV conditions, our data suggest that *elf3* is indeed important for microbially responsive transcriptional programs. We observed attenuation of host immune genes in colonized *elf3* mutants, supporting previous findings that Elf3 regulates cytokine responses to pro-inflammatory stimuli in human bronchial epithelial cells (40) and synovial fibroblasts (37). The attenuated host immune response also included several hematopoietic marker genes, consistent with a finding that *elf3* mediates T cell priming in a murine model of pulmonary inflammation (94). Curiously, *elf3* is required for microbial upregulation of several hematopoietic cell markers despite not being strongly expressed in red blood or innate immune cells under conventionally reared conditions (Fig . S1). Likewise, we find that *elf3* mutation does not have a significant effect on circulating neutrophil numbers in conventionally reared (CR) zebrafish larvae (Fig. 6B). Importantly, as with all other transcriptional differences discussed here, our data do not distinguish between direct or indirect roles for *elf3*. Previous literature indicates that epithelial cells can signal through matrix metalloproteinases to enhance macrophage recruitment in response to *Mycobacterium marinum* (95), and our data set indicates that there is an attenuation of microbial induction of *mmp13a* in *elf3* mutants. It is possible that the *elf3* mutation has direct or indirect effects on epithelial-to-immune cell crosstalk. We also found that *elf3* is required for full expression of other microbially induced TFs (i.e., IRFs) and cytokines that may facilitate the crosstalk between epithelial and other host tissues and the immune system. This aligns with emerging evidence that *elf3* directly binds to the IRF6 promoter in a human gastric cancer cell line (96) and to the interferon epsilon promoter in HEK293 cells (24).

Our findings also suggest there might be *elf3*-dependent transcriptional programs like ECM organization and assembly that are specific to the state of microbial colonization. ECM organization genes were previously demonstrated to be upregulated in conventionally reared *elf3* knockdown larvae compared to controls (25), and we found that ECM organization genes were upregulated in *elf3* mutant larvae compared to wild type only in CV conditions. Not all the demonstrated functions of *elf3*, such as regulation of epithelial-mesenchymal transition seen in numerous human cell culture studies (83–93), exhibited a strong transcriptional signature in our work. This suggests that there might be a degree of genetic redundancy or differences in *elf3* functions across species or certain tissues.

We found that *elf3* mutants exhibit reduced survival fitness as adults despite normal development and expected Mendelian ratios in larvae. One possible explanation for this age-dependent survival phenotype could be age-dependent differences in immunity. Zebrafish do not possess a mature adaptive immune system until approximately 4–6 weeks post-fertilization (97). Our 6 dpf RNA-seq results do not permit evaluation of how *elf3* mutation affects mature adaptive immune responses, which may have important implications for the spontaneous illness and death we observed in *elf3^−/−^* adults. Additional studies are needed to assess the effects of *elf3* mutation on the microbiome of larval zebrafish and adult tissues that may contribute to infection, such as the intestine and swim bladder.

Interestingly, we found that Elf3 is protective against spontaneous inflammation of the swim bladder (aerocystitis), even though *elf3* does not appear to be expressed in the adult swim bladder (98). In larvae, *elf3* is expressed along the gastrointestinal tract, including the esophagus, pharynx, and pneumatic duct (Fig. S1A). A putative cause for the aerocystitis is infectious agents, such as ascending bacteria, that could migrate from the digestive tract through the pneumatic duct to infect the swim bladder. Alternatively, or in concert, reduced function of the pneumatic duct in *elf3* mutants could cause or impair swim bladder buoyancy regulation and subsequently render the duct and swim bladder susceptible to inflammation and infection.

**Fig 7:**
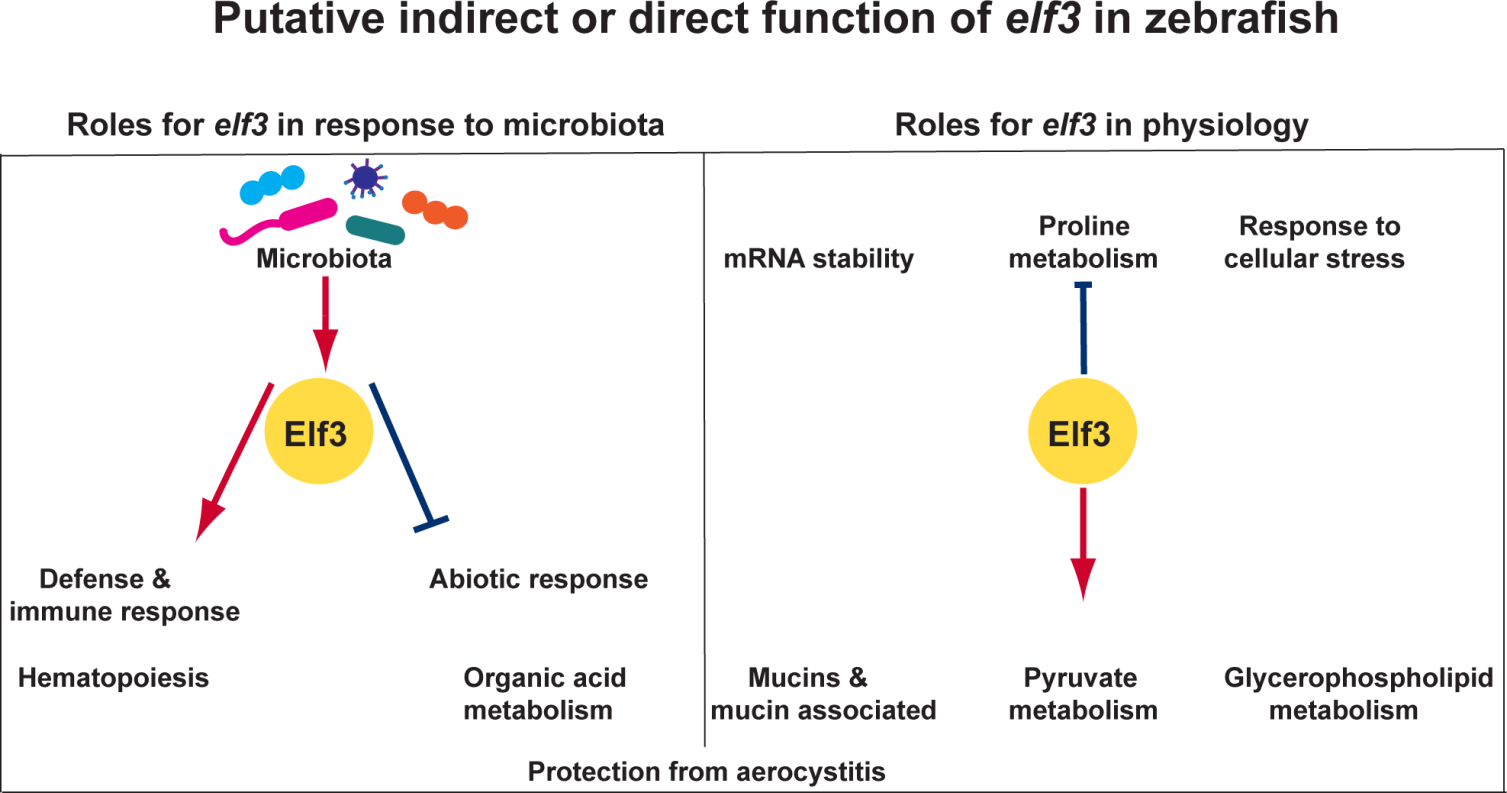
Summary model indicating putative roles for elf3 in zebrafish. *elf3* has roles in mediating host transcriptional responses to the microbiota as well as transcriptional programs important for microbe-independent physiologic functions. In response to microbes, *elf3* mediates the upregulation of genes involved in immune and defense response as well as hematopoietic marker genes while mediating the downregulation of genes with roles in abiotic responses (e.g., circadian rhythm) and organic acid metabolism. Additionally, *elf3* mutation results in the upregulation of transcriptional programs like mRNA stability, proline metabolism, and response to stress and the downregulation of pyruvate and glycerophospholipid metabolism genes in a microbe-independent manner. *elf3* mutation has significant consequences for the survival of adult zebrafish; however, it remains unclear if this effect is related to the role of *elf3* in host-microbiota interactions. All the putative roles for *elf3* indicated in this model could be direct or indirect.

Together, our findings indicate that *elf3* transcriptionally mediates host-microbiota interactions as well as microbe-independent programs important for animal physiology. Our model presents putative direct or indirect roles of *elf3* in the upregulation of immune/defense responses and hematopoiesis while facilitating the downregulation of abiotic responses and organic acid metabolism in response to microbes (Fig. 7). *elf3* also putatively mediates metabolic programs like proline and pyruvate metabolism and cellular stress responses in a microbe-independent manner (Fig. 7). Additional studies are needed to determine if the requirement for *elf3* in protection against aerocystitis is related to its roles in host-microbiota interactions or in microbe-independent programs.

This study establishes that *elf3* transcriptionally mediates host immune responses to the microbiota and is important for adult survival under standard housing conditions. While our results establish putative direct and indirect target genes of *elf3* upon microbial stimulation, additional studies are needed to understand the larger upstream pathways involved in regulating *elf3* expression and its microbial responsiveness as well as direct Elf3 target genes. Our identification of conserved NF-κB binding sites in the *elf3* promoter (Fig. 1) is consistent with previous findings that NF-κB signaling mediates *elf3* expression in diverse cell types (36–40). Upstream of NF-κB, it is also possible that innate immune signaling mediators like toll-like receptors and Myd88 are required for microbial induction of *elf3*. Previous studies investigating host responses to conventional (99) and pathogenic exposures (29) in larval zebrafish suggest that *myd88* may mediate the microbial induction of *elf3*. Future genetic or pharmacological studies that directly test the ability of these TFs to regulate *elf3* will provide additional context to its role mediating host-microbiota interactions.

## MATERIALS AND METHODS

### Zebrafish husbandry

Husbandry of conventionally reared zebrafish was performed as described (100, 101). Briefly, zebrafish stocks maintained on an Ekkwill or a mixed EK/Tübingen long fin background were housed on a Pentair recirculating aquaculture system with a 14/10 hour light/dark cycle at 28°C. Generation and maintenance of gnotobiotic larvae was performed as previously described (10), with the addendum that the “antibiotic-containing gnotobiotic zebrafish medium” was supplemented with 50 µg/mL gentamicin (Sigma-Aldrich, G1264) (100). Genotyping of larval and adult zebrafish was performed using standard whole larvae preparations or fin tissue resection methods, with the extracted genomic DNA amplified using the primers listed in Table S1. For imaging experiments, fish were anesthetized in buffered tricaine prior to mounting.

### Generation of mutant zebrafish alleles

Mutagenesis of the *elf3* locus was performed with CRISPR/Cas9-based genome editing as described (44). Briefly, the “CRISPRscan” tool (https://www.crisprscan.org/) was used to identify the target sequence of both guide RNAs (gRNAs). For *elf3^rdu102^,* the gRNA was synthesized via *in vitro* transcription. One- to two-cell-stage wild-type embryos were injected with a mixture of gRNA (120 ng/µL), Cas9 mRNA (150 ng/µL), 0.05% phenol red, 120 mM NaCl, and 20 mM HEPES buffer (pH 7.0). For *elf3^rdu103^,* a synthesized gRNA targeting the DNA-binding domain was purchased from IDTDNA. One- to two-cell-stage wild-type embryos were injected with a mixture of this gRNA (80 ng/µL), Cas9 protein (300 ng/µL), and 0.06% phenol red. For both alleles, mutagenesis events in injected embryos were detected via heteroduplex mobility assay. Founders for both alleles were identified via Sanger sequencing of PCR products that spanned the CRISPR target site. *elf3^rdu102^* was identified as a 19 bp deletion, which is predicted to result in a premature stop codon after amino acid 235 (Fig. S2). *elf3*^rdu103^ was identified as a 12 bp in-frame deletion (Fig. 2). Founders were outcrossed to wild-type fish to propagate both alleles and generate stable lines. *elf3*^rdu103^ is the primary allele characterized in this paper. Genotyping primers and gRNA sequences are listed in Table S1.

### Statistical methods

All statistical tests, excluding the bioinformatic analyses of the bulk RNA-seq data, were generated with GraphPad Prism 10 statistical software. *P*-values generated from these statistical tests are indicated in the figure or figure legend. *P* < 0.05 was used to determine statistically significant findings. Data are presented as mean ± SEM, unless otherwise indicated.

### Additional methods

Additional methods for RNA extraction, bulk RNA sequencing and bioinformatic analysis, brightfield imaging, longitudinal assessment of *elf3* mutant survival, histopathology of adult zebrafish, larval zebrafish infections with *Mycobacterium marinum*, and isolation and identification of swim bladder bacteria are available in Text S1 in the supplemental material.

## Data Availability

Raw sequencing and count data for the bulk RNA-seq can be accessed via the NCBI’s Genome Expression Omnibus through GEO Series accession number GSE293676.

## SUPPLEMENTAL MATERIAL

Supplemental Material mbio.02267-25-s0001.pdf Supplemental methods and Fig. S1 to S6.

Table S1 mbio.02267-25-s0002.xlsx Common reagents used in the study.

Table S2 mbio.02267-25-s0003.xlsx HOMER TFBS motif enrichment results analyzing the accessible chromatin near microbially responsive genes.

Table S3 mbio.02267-25-s0004.xlsx Published mouse and zebrafish studies used to compare CV/GF expression.

Table S4 mbio.02267-25-s0005.xlsx RNA-seq differential gene expression analysis.

Table S5 mbio.02267-25-s0006.xlsx Expression of zebrafish developmental genes identified in Yang et al. 2013.

Table S6 mbio.02267-25-s0007.xlsx Functional enrichment analysis for significant differentially expressed genes in RNA-seq.

ASM does not own the copyrights to Supplemental Material that may be linked to, or accessed through, an article. The authors have granted ASM a non-exclusive, world-wide license to publish the Supplemental Material files. Please contact the corresponding author directly for reuse.
