# Supplementary material for: Epithelial transcription factor Elf3 mediates host immune responses to microbiota and protects against aerocystitis in zebrafish
Source: mBio. 2025 Oct 28;16(12):e02267-25. doi: 10.1128/mbio.02267-25 (PMC12691682; doi:10.1128/mbio.02267-25)
Supplement: Supplemental Material — Supplemental methods and Fig. S1 to S6. [file mbio.02267-25-s0001.pdf]

Epithelial transcription factor Elf3 mediates host immune responses to microbiota and protects against aerocystitis in zebrafish

Briana R. Davis[a], Colin R. Lickwar[a], Christiane V. Löhr[b], Jia Wen[a], Margaret Morash[a], Mollie I. Sweeney[a], Elizabeth L. Reich[a], Peyton J. Moore[a], David M. Tobin[a,c], and John F. Rawls[a,*]

[a]Department of Molecular Genetics and Microbiology, Duke University School of Medicine, Durham, NC 27710, USA
[b]Oregon Veterinary Diagnostic Laboratory, Carlson College of Veterinary Medicine, Oregon State University, Corvallis, OR 97331, USA
[c]Department of Integrative Immunobiology, Duke University School of Medicine, Durham, NC 27710, USA

* Address correspondence to John F. Rawls, john.rawls@duke.edu

SUPPLEMENTAL MATERIALS

Text S1: Supplemental methods

RNA extraction for larval bulk RNA sequencing: *elf3*$^{+/+}$ and *elf3*^{rdu103} larvae were pooled separately from at least two clutches of homozygous incrosses from parental cousins. Larvae were euthanized in buffered Tricaine (n= 6-16 pooled larvae per experimental replicate) and stored at 4°C in RNAlater (Thermo Fisher, AM7020) for 16 hours prior to long-term storage at –80°C. To start the RNA extraction process, the larvae were washed in 1 mL of cold GZM. Next, 1 mL of TRIzol (Thermo Fisher, 15596026) was added to each replicate tube, and the larvae were homogenized with a 27-gauage needle and syringe (25 total passes). Following homogenization, the RNA extraction process was completed with the PureLink RNA Mini Kit (Invitrogen, 12183018A) according to the manufacturer's instructions. To ensure high quality RNA for sequencing, the eluted RNA was prepared for additional clean up with the Zymo RNA Clean and Concentrator Kit (Zymo Research, R1017). Total RNA was stored at –80°C until submission to the Duke Sequencing and Genomic Technologies Shared Resource (SGT). The SGT Core generated stranded mRNA libraries (KAPA HyperPrep) and performed 150 bp paired-end sequencing in one 10B lane of the NovaSeq X Plus.

Bulk RNA-sequencing bioinformatic analysis: Raw sequencing files were uploaded to the Galaxy server, where the paired reads were subsequently trimmed using Trim Galore! to remove adapter sequences and low-quality reads. These trimmed reads were then mapped to the *Danio rerio* reference genome (GRCz11) with default STAR RNA-seq (1) parameters using the Lawson lab transcriptome annotations as a gene model (v4.3.2 GTF) (2). Mapped reads per gene were quantified via FeatureCounts with default parameters (3) and the resulting counts for each replicate were used to compute

differential expression analysis and likelihood ratio test (LRT) via DESeq2 (4, 5). Deseq2 and its plotPCA function were used to generate the PCA plot of normalized counts for each replicate. The Venn diagram of significant differentially expressed genes was generated online via (http://bioinformatics.psb.ugent.be/webtools/Venn/). To identify GO biological processes and other pathways enriched in our lists of differentially expressed genes, we used the default settings of Metascape (6).

Brightfield and color camera imaging of zebrafish: To generate brightfield images of larval zebrafish, anesthetized fish were mounted in 3% methylcellulose [w/v in gnotobiotic zebrafish media (GZM0000)] and imaged with a Leica DFC 365FX camera attached to a Leica M205 FA stereomicroscope. Colored images of anesthetized adult zebrafish were taken with a Leica DFC425 camera attached to a Nikon SMT-2T dissecting scope.

Longitudinal assessment of *elf3*^{rdu103} survival: We in-crossed *elf3*^{rdu103} heterozygous adults and separately in-crossed *elf3*$^{+/+}$ and *elf3*$^{-/-}$ siblings to generate the heterozygous (n = 4 tanks) and homozygous (n = 2 tanks per genotype) that we observed over time. For the heterozygous tanks, we genotyped the fish with standard fin resection methods at 60 dpf to obtain the starting genotypic ratios and performed genotyping rounds at 100 dpf, 277 dpf, and 368 dpf to monitor ratios. For the homozygous tanks, we performed tank counts at the same timepoints that the genotyping was conducted with 4 additional timepoints (130 dpf, 194 dpf, 232 dpf, 313 dpf). All survival data is presented in the form of Kaplan Meier survival curves. A death event was defined as any fish that was found dead, euthanized for a humane endpoint, or fish presumed dead resulting from decreased "n" at time of tank count. Moribund fish presenting clinical signs of health deterioration such as abnormal swimming, ulcers, and erythema were euthanized for a humane endpoint. Several of these euthanized moribund fish were prepared for adult histopathological assessment. Wild-type controls that were euthanized specifically to serve as controls for the histopathological assessment were considered censored and are indicated as such in the Kaplan Meier survival curve as ticks.

Histopathologic evaluation of adult zebrafish: Moribund *elf3* mutant adults ranging from 202 to 501 dpf were euthanized along with sex-matched, similarly aged wild-type controls (≤ 20 dpf difference) in preparation for paired analysis. For the cross-sectional cohort, mutant and wild-type adults (7 males and 7 females per genotype) were euthanized at 154 dpf. Euthanized fish were prepared for whole animal histology according to the ZIRC Public Wiki fixation protocol (www.zebrafish.org) with the addendum that the de-calcified fish were rinsed for 3 hours in running DI water before immersion in a graded ethanol series ranging from 25% to 70%. The fish were stored in 70% ethanol prior to shipping to the Oregon Veterinary Diagnostic Laboratory at Oregon State University, where the fish were embedded in paraffin blocks, sectioned in a paramedian plane, and stained to produce hematoxylin and eosin (H&E), acid fast, and Gram-stained slides. The Duke BioRepository and Precision Pathology Center (BRPC) generated 40X whole slide scans using a Leica Aperio GT450 scanner. Histopathologic evaluation of the scanned slides was performed in a blinded, paired analysis by a board-certified veterinary pathologist. For consistency, swim bladder histology images depicted in the publication were either rotated or vertically reflected for head left orientation.

Larval zebrafish infections with *Mycobacterium marinum*: 2 dpf larval zebrafish from homozygous incrosses of *elf3* wild-type and homozygous mutant parents were anesthetized with tricane and approximately 200 fluorescent bacteria (FB) of wild-type, red fluorescent *M. marinum* were injected into the caudal vein of each larval zebrafish with a borosilicate needle. The infected zebrafish were subsequently recovered in E3 medium containing 1-phenyl 2-thiourea (PTU). Any embryos damaged in the process of injection or infected with incorrect dosage were removed from the experiment at 1 dpi. Final images were taken at 5 dpi. Fluorescent bacteria were quantified as previously described (7).

Isolation and identification of swim bladder bacteria: To evaluate swim bladder associated infection in *elf3* mutant zebrafish, we selected moribund mutant fish that were displaying aberrant swimming behavior and wild-type control siblings (n = 6 mutants and n = 4 wild-type). Immediately following euthanasia in buffered Tricaine, the fish were soaked in 10% PVP-iodine (Ricca 3955-16) for 2 minutes, gently scrubbed with PVP-iodine soaked kimwipes, and re-submerged in the iodine solution for an additional 2 minutes. Swim bladders were dissected with sterile instruments, homogenized with a pestle, and plated on blood agar plates (tryptic soy agar base with 5% sheep blood, VWR 10324-332) under aerobic and anaerobic conditions. Cultured bacteria were isolated and their 16S rRNA genes were PCR amplified (Table S1), Sanger sequenced and classified using SILVA (8-10).

Enumeration of lyz:EGFP$^+$ cells in larval zebrafish: 6 dpf *elf3* wild-type and mutant larvae were anesthetized using Tricaine, mounted in 3% methylcellulose, and imaged with a Leica DFC 365FX camera attached to a Leica M205 FA stereomicroscope. Neutrophils in the larvae were quantified using an automated FIJI macro. First, three representative images were used to set the parameters of the macro, including background signal reduction and thresholding methods. Parameters were chosen to optimize detection of neutrophils with the least detection of background or autofluorescence signal. For each image, background signal was first subtracted (rolling ball radius = 5.0 pixels), followed by automatic thresholding using the MaxEntropy method. The thresholded image was then converted to a binary file and subjected to the watershed function to declare if single particles were likely representative of two or more cells. Finally, the analyze particle's function (size micron^2 = 10-infinity, circularity = 0.00-1.00) was run to calculate number of detected cells. The output of this macro reports the number of *lyz*+ neutrophils per whole larvae (rawCount) and the area of each cell. An R script was also used to determine the median area of the detected particles within a single fish, divide the area of each particle by this value to determine if one particle is representative of more than one cell, then report in a new column the number of cells based on this measurement (medCount). The medCount is what is presented in Fig.S6B.

References

1. Dobin A, Davis CA, Schlesinger F, Drenkow J, Zaleski C, Jha S, Batut P, Chaisson M, Gingeras TR. 2013. STAR: ultrafast universal RNA-seq aligner. Bioinformatics 29:15-21.
2. Lawson ND, Li R, Shin M, Grosse A, Yukselen O, Stone OA, Kucukural A, Zhu L. 2020. An improved zebrafish transcriptome annotation for sensitive and comprehensive detection of cell type-specific genes. Elife 9.
3. Liao Y, Smyth GK, Shi W. 2014. featureCounts: an efficient general purpose program for assigning sequence reads to genomic features. Bioinformatics 30:923-30.
4. Love MI, Huber W, Anders S. 2014. Moderated estimation of fold change and dispersion for RNA-seq data with DESeq2. Genome Biol 15:550.
5. Lickwar CR, Davison JM, Kelly C, Mercado GP, Wen J, Davis BR, Tillman MC, Semova I, Andres SF, Vale G, McDonald JG, Rawls JF. 2022. Transcriptional Integration of Distinct Microbial and Nutritional Signals by the Small Intestinal Epithelium. Cell Mol Gastroenterol Hepatol 14:465-493.
6. Zhou Y, Zhou B, Pache L, Chang M, Khodabakhshi AH, Tanaseichuk O, Benner C, Chanda SK. 2019. Metascape provides a biologist-oriented resource for the analysis of systems-level datasets. Nat Commun 10:1523.
7. Takaki K, Davis JM, Winglee K, Ramakrishnan L. 2013. Evaluation of the pathogenesis and treatment of Mycobacterium marinum infection in zebrafish. Nat Protoc 8:1114-24.
8. Quast C, Pruesse E, Yilmaz P, Gerken J, Schweer T, Yarza P, Peplies J, Glockner FO. 2013. The SILVA ribosomal RNA gene database project: improved data processing and web-based tools. Nucleic Acids Res 41:D590-6.
9. Yilmaz P, Parfrey LW, Yarza P, Gerken J, Pruesse E, Quast C, Schweer T, Peplies J, Ludwig W, Glockner FO. 2014. The SILVA and "All-species Living Tree Project (LTP)" taxonomic frameworks. Nucleic Acids Res 42:D643-8.
10. Glockner FO, Yilmaz P, Quast C, Gerken J, Beccati A, Ciuprina A, Bruns G, Yarza P, Peplies J, Westram R, Ludwig W. 2017. 25 years of serving the community with ribosomal RNA gene reference databases and tools. J Biotechnol 261:169-176.
11. Montfort J, Hervas-Sotomayor F, Le Cam A, Murat F. 2024. FEVER: an interactive web-based resource for evolutionary transcriptomics across fishes. Nucleic Acids Res 52:W65-W69.
12. Sur A, Wang Y, Capar P, Margolin G, Prochaska MK, Farrell JA. 2023. Single-cell analysis of shared signatures and transcriptional diversity during zebrafish development. Dev Cell 58:3028-3047 e12.
13. Yang H, Zhou Y, Gu J, Xie S, Xu Y, Zhu G, Wang L, Huang J, Ma H, Yao J. 2013. Deep mRNA sequencing analysis to capture the transcriptome landscape of zebrafish embryos and larvae. PLoS One 8:e64058.
14. Massaquoi MS, Kong GL, Chilin-Fuentes D, Ngo JS, Horve PF, Melancon E, Hamilton MK, Eisen JS, Guillemin K. 2023. Cell-type-specific responses to the microbiota across all tissues of the larval zebrafish. Cell Rep 42:112095.

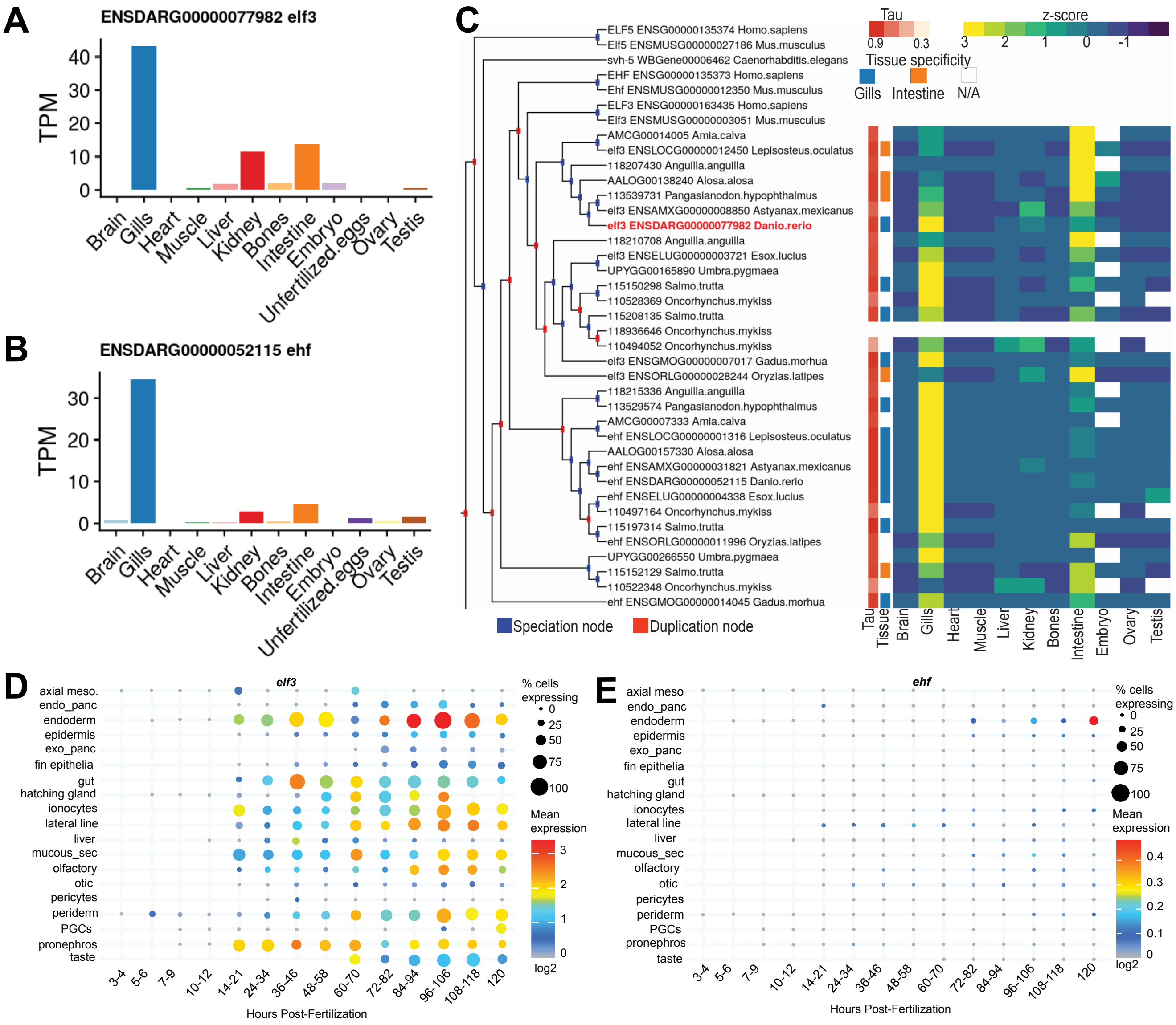

A
ENSDARG00000077982 elf3
TPM
0
10
20
30
40
Brain
Gills
Heart
Muscle
Liver
Kidney
Bones
Intestine
Embryo
Unfertilized.eggs
Ovary
Testis
B
ENSDARG00000052115 ehf
TPM
0
10
20
30
Brain
Gills
Heart
Muscle
Liver
Kidney
Bones
Intestine
Embryo
Unfertilized.eggs
Ovary
Testis
C
ELF5 ENSG00000135374 Homo.sapiens
Elf5 ENSMUSG00000027186 Mus.musculus
svh-5 WBGene00006462 Caenorhabditis.elegans
EHF ENSG00000135373 Homo.sapiens
Ehf ENSMUSG00000012350 Mus.musculus
ELF3 ENSG00000163435 Homo.sapiens
Elf3 ENSMUSG00000003051 Mus.musculus
AMCG00014005 Amia.calva
elf3 ENSLOCG00000012450 Lepisosteus.oculatus
118207430 Anguilla.anguilla
AALOG00138240 Alosa.alosa
113539731 Pangasianodon.hypophthalmus
elf3 ENSAMXG00000008850 Astyanax.mexicanus
elf3 ENSDARG00000077982 Danio.rerio
118210708 Anguilla.anguilla
elf3 ENSELUG00000003721 Esox.lucius
UPYGG00165890 Umbra.pygmaea
115150298 Salmo.trutta
110528369 Oncorhynchus.mykiss
115208135 Salmo.trutta
118936646 Oncorhynchus.mykiss
110494052 Oncorhynchus.mykiss
elf3 ENSGMOG00000007017 Gadus.morhua
elf3 ENSORLG00000028244 Oryzias.latipes
118215336 Anguilla.anguilla
113529574 Pangasianodon.hypophthalmus
AMCG00007333 Amia.calva
ehf ENSLOCG00000001316 Lepisosteus.oculatus
AALOG00157330 Alosa.alosa
ehf ENSAMXG00000031821 Astyanax.mexicanus
ehf ENSDARG00000052115 Danio.rerio
ehf ENSELUG00000004338 Esox.lucius
110497164 Oncorhynchus.mykiss
115197314 Salmo.trutta
ehf ENSORLG00000011996 Oryzias.latipes
UPYGG00266550 Umbra.pygmaea
115152129 Salmo.trutta
110522348 Oncorhynchus.mykiss
ehf ENSGMOG00000014045 Gadus.morhua
Speciation node
Duplication node
Tau
0.9
0.3
z-score
3
2
1
0
-1
Tissue specificity
Gills
Intestine
N/A
Tau
Tissue
Brain
Gills
Heart
Muscle
Liver
Kidney
Bones
Intestine
Embryo
Ovary
Testis
D
elf3
axial meso.
endo_panc
endoderm
epidermis
exo_panc
fin epithelia
gut
hatching gland
ionocytes
lateral line
liver
mucous_sec
olfactory
otic
pericytes
periderm
PGCs
pronephros
taste
3-4
5-6
7-9
10-12
14-21
24-34
36-46
48-58
60-70
72-82
84-94
96-106
108-118
120
Hours Post-Fertilization
% cells expressing
0
25
50
75
100
Mean expression
3
2
1
0
log2
E
ehf
axial meso
endo_panc
endoderm
epidermis
exo_panc
fin epithelia
gut
hatching gland
ionocytes
lateral line
liver
mucous_sec
olfactory
otic
pericytes
periderm
PGCs
pronephros
taste
3-4
5-6
7-9
10-12
14-21
24-34
36-46
48-58
60-70
72-82
84-94
96-106
108-118
120
Hours Post-Fertilization
% cells expressing
0
25
50
75
100
Mean expression
0.4
0.3
0.2
0.1
0
log2

Figure S1. ***elf3* is expressed in diverse tissues in adult and larval zebrafish.** Bar plots generated by the FEVER transcriptomics web server (11) that show the expression in transcripts per million (TPM) for *elf3* (**A**) and *ehf* (**B**) in adult zebrafish tissues. (**C**) FEVER evolutionary gene tree with blue duplication and red speciation nodes for ESE ETS TFs across 13 fishes and corresponding heatmap of z-scored gene expression in 11 tissues. The FEVER-calculated tissue-specificity indexes (Tau) are calculated for genes with no missing expression values and indicate high specificity when >0.9. Genes with Tau specificity (>0.9) for the gills and intestine are indicated by the blue and orange boxes respectively. (**D-E**) Dot plot visualization from the Daniocell (12) scRNA-seq atlas of larval zebrafish showing, tissue-specific expression of *elf3* and *ehf* at the indicated developmental timepoints. Abbreviations: axial meso = axial mesoderm, endo_panc = endocrine pancreas; exo_pan = exocrine pancreas, mucous_sec = mucous secreting, PGCs: primordial germ cells.

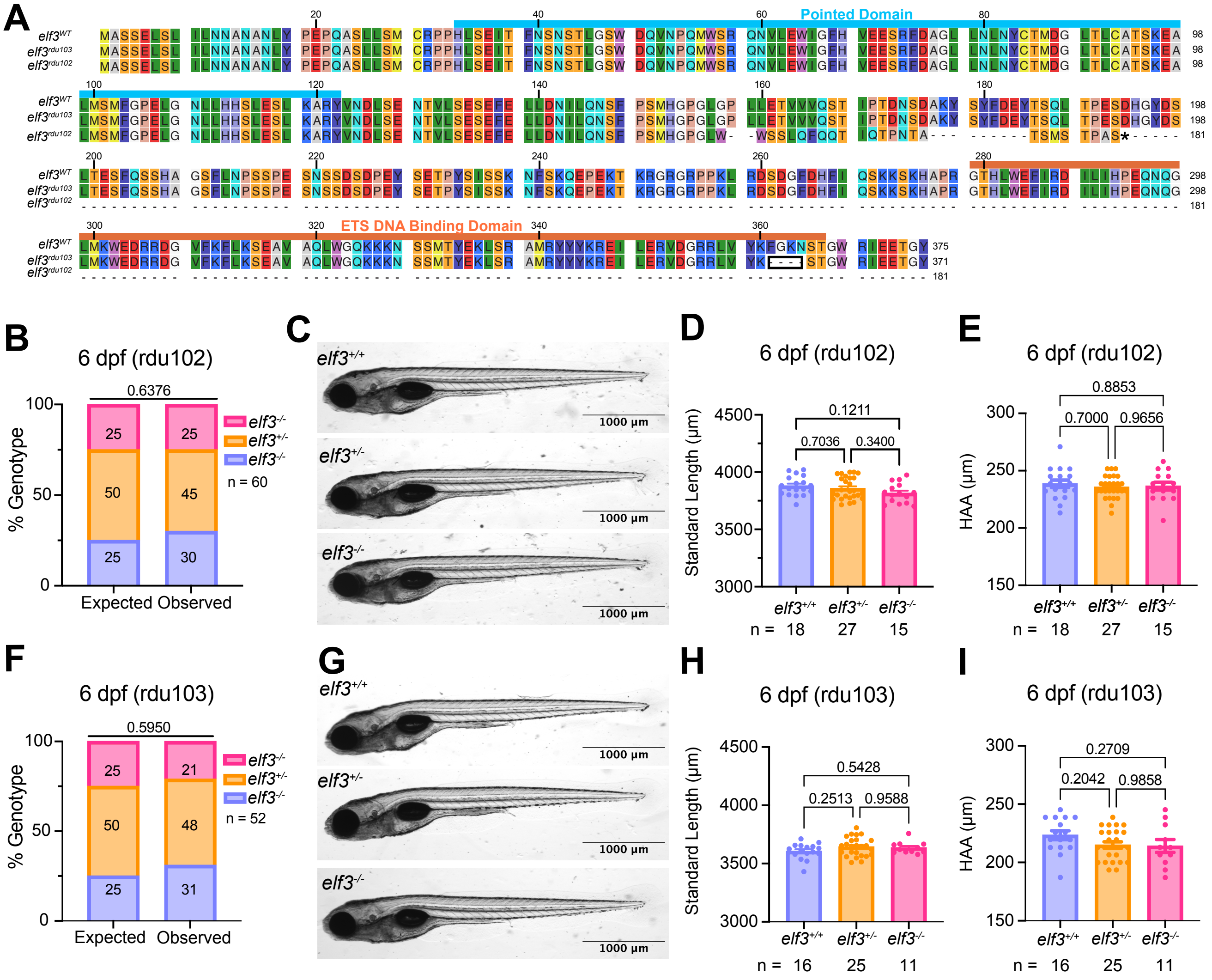
A
Pointed Domain
ETS DNA Binding Domain
elf3WT
elf3rdu103
elf3rdu102
B
6 dpf (rdu102)
0.6376
% Genotype
Expected Observed
elf3-/-
elf3+/-
elf3+/+
n = 60
C
elf3+/+
elf3+/-
elf3-/-
1000 µm
D
6 dpf (rdu102)
Standard Length (µm)
0.1211
0.7036
0.3400
n = 18 27 15
E
6 dpf (rdu102)
HAA (µm)
0.8853
0.7000
0.9656
n = 18 27 15
F
6 dpf (rdu103)
0.5950
% Genotype
Expected Observed
n = 52
G
elf3+/+
elf3+/-
elf3-/-
1000 µm
H
6 dpf (rdu103)
Standard Length (µm)
0.5428
0.2513
0.9588
n = 16 25 11
I
6 dpf (rdu103)
HAA (µm)
0.2709
0.2042
0.9858
n = 16 25 11

Figure S2. Generation of *elf3* mutant alleles. (**A**) Alignment of the wild-type Elf3 protein sequence (ENSDARP00000101728.2) to the predicted mutated sequence for *elf3*^{rdu102} and *elf3*^{rdu103} alleles. The asterisk indicates the early stop codon of the *elf3*^{rdu102} frameshift mutation, and the black box highlights the 4 deleted amino acid residues of the *elf3*^{rdu103} 12 bp in-frame mutation. The predicted pointed and DNA-binding functional domains are indicated by the horizontal blue and orange lines respectively. **(B)** Comparison of the observed genotypic ratios from heterozygous in-cross of *elf3*^{rdu102} adults to expected Mendelian ratios. (**C**) Representative brightfield images of *elf3*^{rdu102} allele 6 dpf larvae. (**D-E**) Standard length (snout to caudal peduncle) and height at anterior of anal fin (HAA) of *elf3*^{rdu102} larvae with n per genotype (+/+: 18, +/-: 27, -/-:15). (**F**) Comparison of the observed genotypic ratios from heterozygous in-cross of *elf3*^{rdu103}adults to expected Mendelian ratios. (**G**) Representative brightfield images of *elf3*^{rdu103} 6 dpf larvae. (**H-I**) Morphometric analysis of the standard length (snout to caudal peduncle) and height at anterior of anal fin (HAA) of *elf3*^{rdu103} with n per genotype (+/+: 16, +/-: 25, -/-:11). Data are presented as mean ± SEM. *P*-values for (**B & F**) were calculated using a chi-square goodness-of-fit test and (**D-E & H-I**) using a one-way ANOVA with Tukey's multiple comparisons post-test.

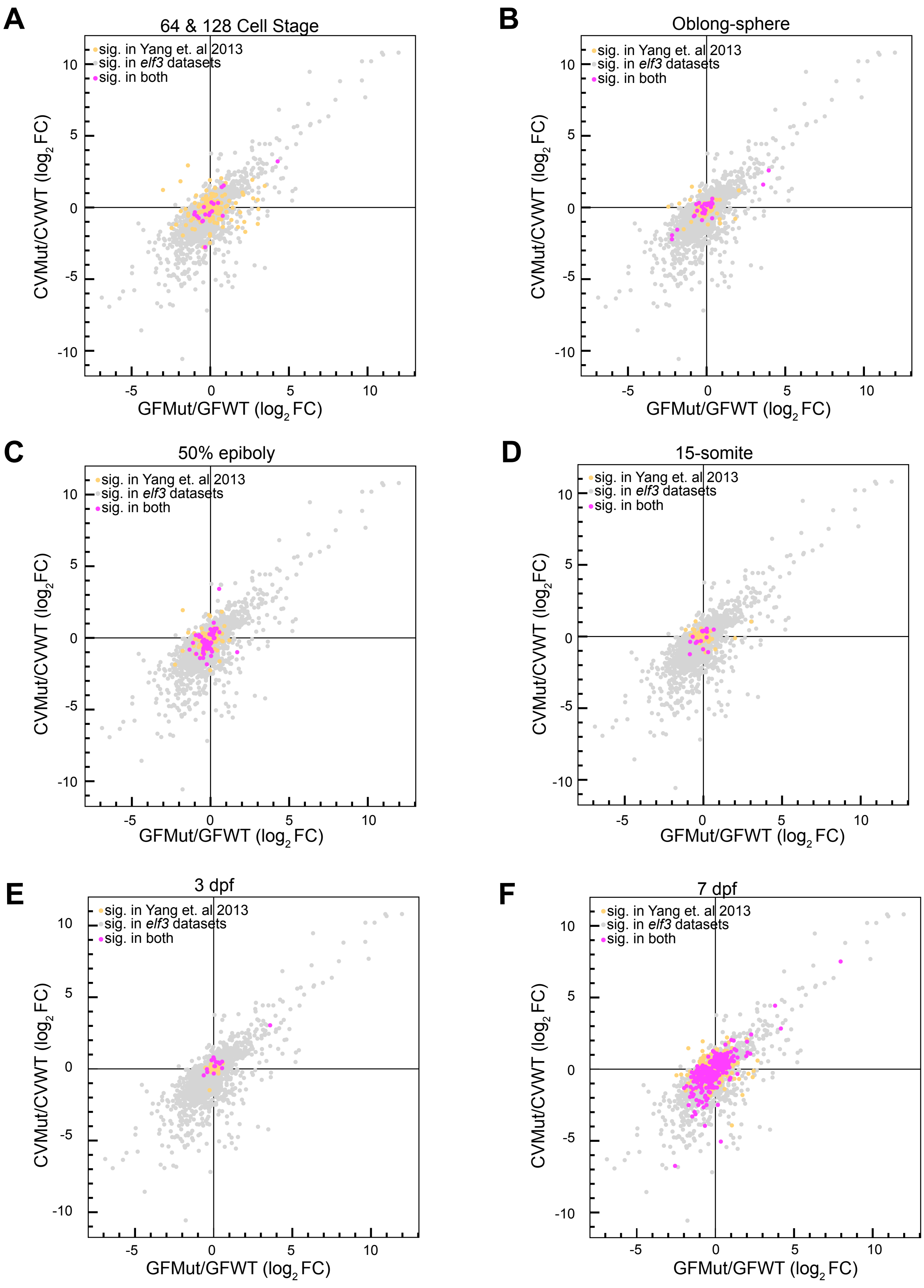
A
64 & 128 Cell Stage
B
Oblong-sphere
C
50% epiboly
D
15-somite
E
3 dpf
F
7 dpf
sig. in Yang et. al 2013
sig. in elf3 datasets
sig. in both
CVMut/CVWT (log_2 FC)
GFMut/GFWT (log_2 FC)
10
5
0
-5
-10

Figure S3. *elf3* mutant larvae are not developmentally delayed. (**A-F**) Scatter plots of developmentally regulated genes overlayed on the larval *elf3* RNA-seq datasets (CVMut/CVWT and GFMut/GFWT comparison). Stages include (**A**) 64 & 128 cell stage, (**B**) oblong-sphere, (**C**) 50% epiboly, (**D**) 15-somite, (**E**) 3 dpf, and (**F**) 7 dpf (13) genes are colored yellow: developmental marker genes, gray: *elf3* datasets, and pink: significant in both. Additional information about the significant genes for developmental timepoint can be found in Table S5.

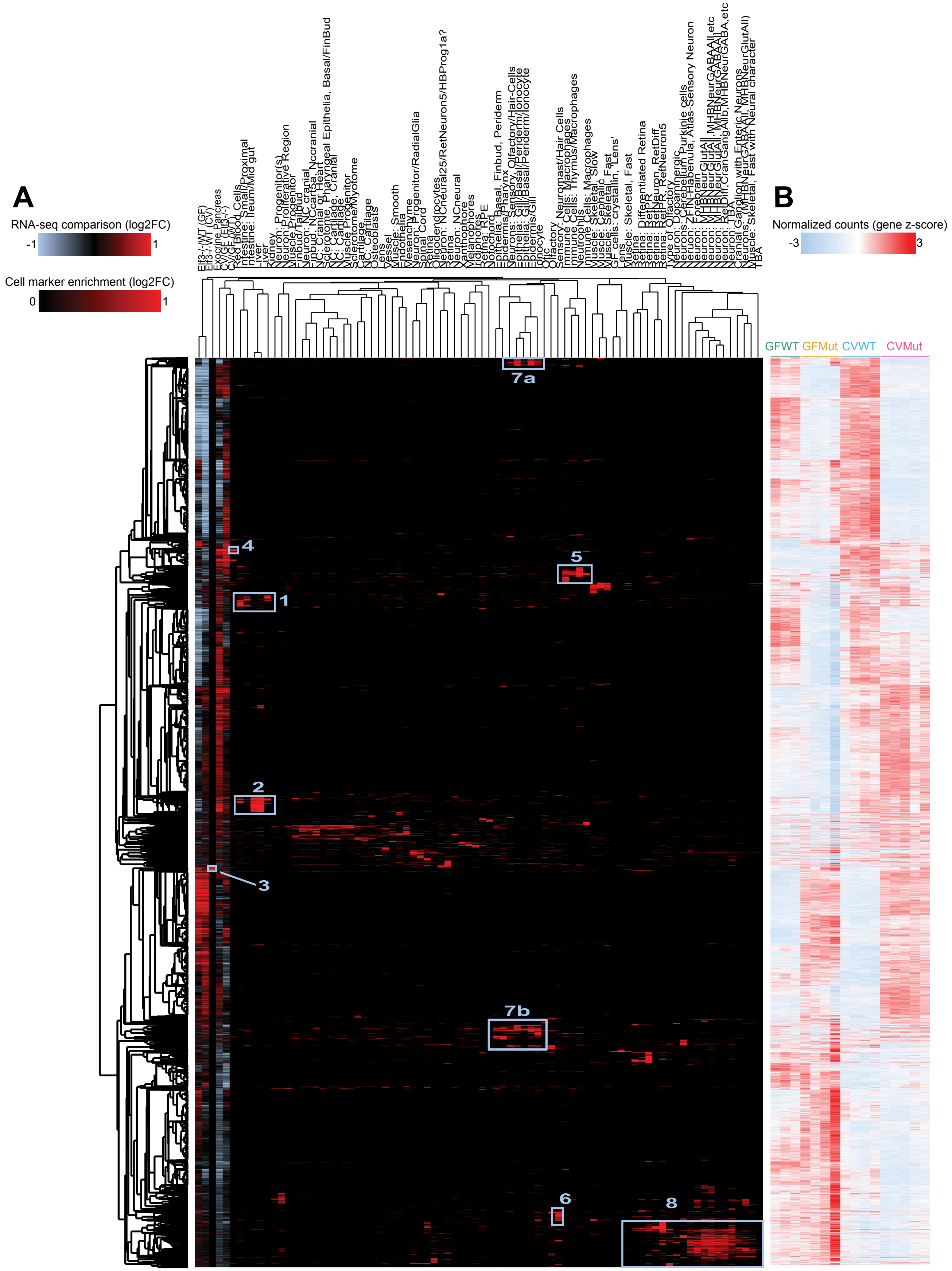
A
RNA-seq comparison (log2FC)
-1
1
Cell marker enrichment (log2FC)
0
1
B
Normalized counts (gene z-score)
-3
3
GFWT
GFMut
CVWT
CVMut
7a
4
5
1
2
3
7b
6
8

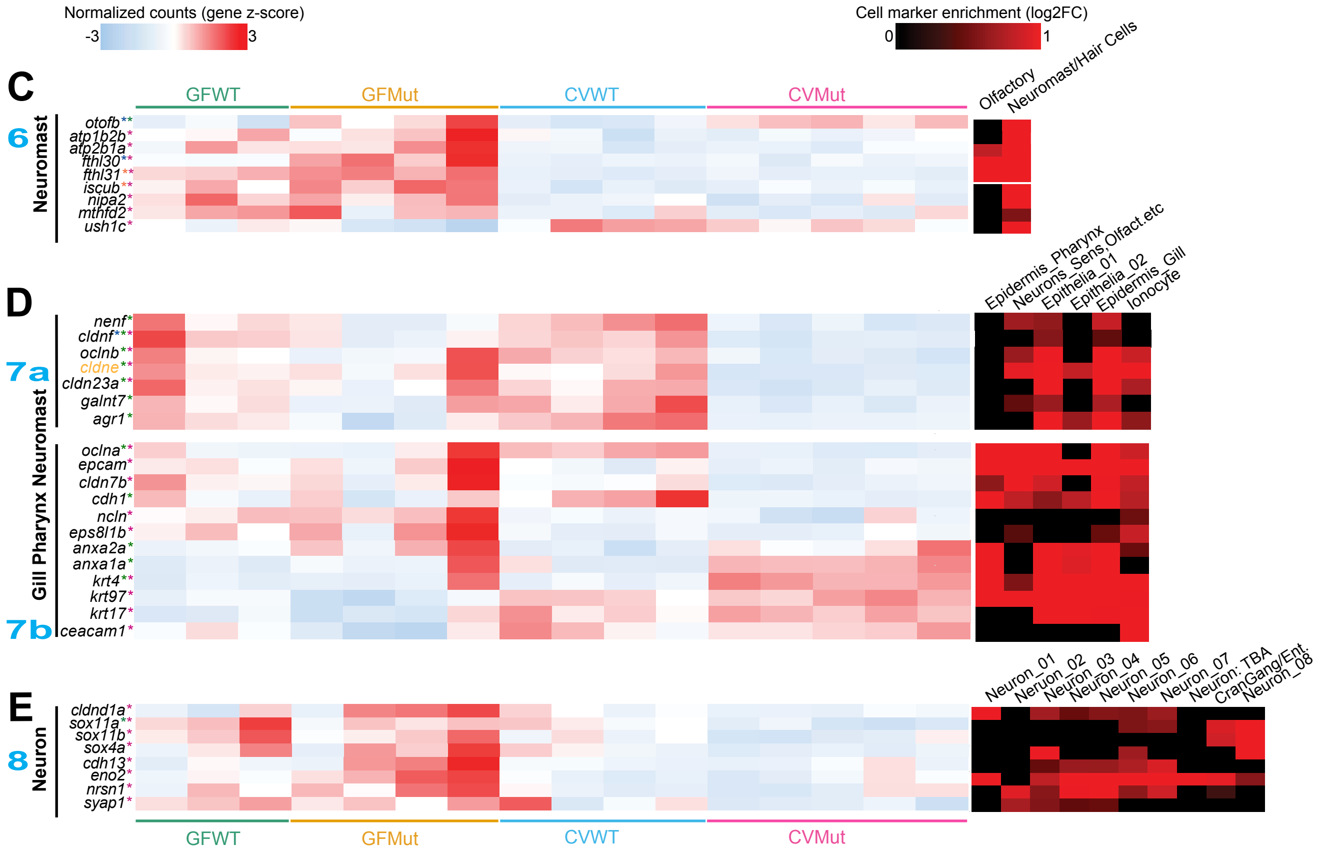

Normalized counts (gene z-score)
-3
3
Cell marker enrichment (log2FC)
0
1
C
GFWT
GFMut
CVWT
CVMut
Olfactory
Neuromast/Hair Cells
6
Neuromast
otofb**
atp1b2b*
atp2b1a*
fthl30**
fthl31**
iscub**
nipa2*
mthfd2*
ush1c*
D
Epidermis_Pharynx
Neurons_Sens,Olfact.etc
Epithelia_01
Epithelia_02
Epidermis_Gill
Ionocyte
7a
7b
Gill Pharynx Neuromast
nenf*
cldnf***
oclnb**
cldne**
cldn23a**
galnt7*
agr1*
oclna**
epcam*
cldn7b*
cdh1*
ncln*
eps8l1b*
anxa2a*
anxa1a*
krt4**
krt97*
krt17*
ceacam1*
E
Neuron_01
Neruon_02
Neuron_03
Neuron_04
Neuron_05
Neuron_06
Neuron_07
Neuron: TBA
CranGang/Ent.
Neuron_08
8
Neuron
cldnd1a*
sox11a**
sox11b*
sox4a*
cdh13*
eno2*
nrsn1*
syap1*
GFWT
GFMut
CVWT
CVMut

Figure S4. Identification of putative cell-type specific responses among differentially expressed genes in larval RNA-seq. (**A**) Clustered heatmap of the $\log_2$ fold change of all significant differentially expressed genes in the RNA-seq analysis of this study plotted with the $\log_2$ fold change of cell type marker genes identified in a previous scRNA-seq dataset (14) uses disassociated cells from whole 6 dpf zebrafish. The blue boxes and associated numbers indicate clusters of genes that are commonly markers for the same cell types and are usually similarly differentially expressed across comparisons in the RNA-seq analysis. (**B**) Heatmap of the gene z-scored normalized counts for all significant differentially expressed genes in at least 1 of the 4 RNA-seq comparisons (P adjusted < .05) ordered by the genes in A. (**C-E**) Heatmaps of z-scored normalized counts for example (**C**) neuromasts, (**D**) gill/pharynx/neuromast tissue and (**E**) neuronal marker genes. All genes in the heatmap are significant in at least 1 RNA-seq comparison. The asterisk next to each gene is colored to indicate which comparison(s) the gene is significantly differential (CVMut/GFMut: maroon, CVWT/GFWT: orange, GFMut/GFWT: blue, and CVMut/CVWT: green). The blue numbers to the left of each blue, white, and red heatmap indicates the specific cell type cluster the example genes represent (Fig. S4A). The red and black heatmaps show the $\log_2$ fold change enrichment of each gene for the indicated cell type (14). Abbreviations: neuron_01 = Cerebellum Purkinje cells; neuron_02 = ZFIN-Habenula/Atlas-Sensory Neuron; neuron_03 = forebrain; neuron_04 = MHBNeurGlutAll; neuron_05 = MHBNeurGlutAII/MHBNeurGABAAII/Cranial ganglion; neuron_06 = MHBNeurGlutAII/MHBNeurGABAAII; neuron_07 = RetDiff/CranGangAIIb, MHBNeurGABA/GlutAII/SCDiff; CranGang/Ent = Cranial Ganglion with Enteric Neurons, neuron_8 = MHBNeurGABAAII, MHBNeurGlutAII; neurons_sens, olfact, etc = neurons/sensory/olfactory/hair cells; epithelia_01 = epithelial: gill/basal/periderm/ionocyte; epithelia_02: epithelial: gill/basal/periderm/ionocyte

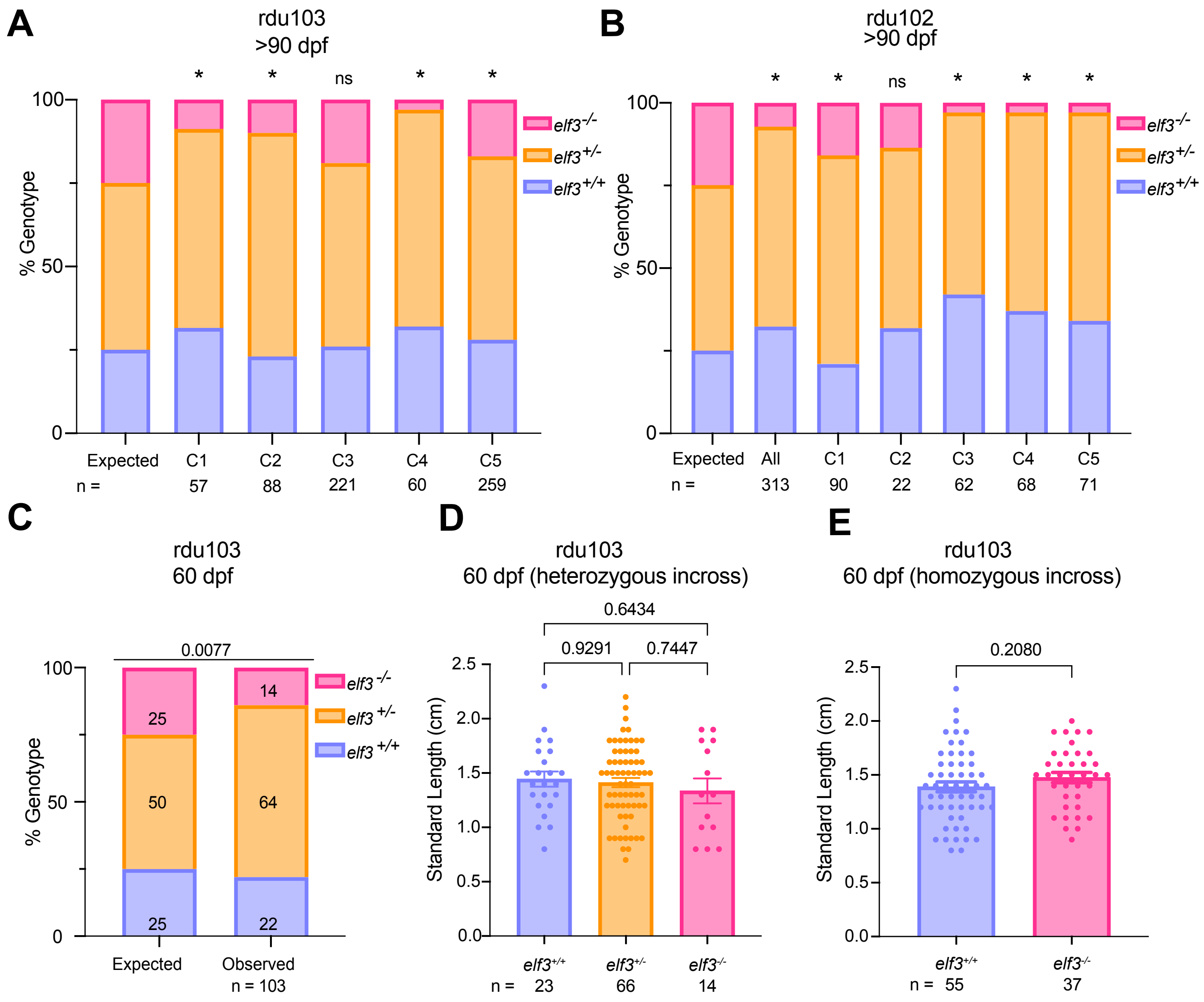
A
rdu103
>90 dpf
% Genotype
elf3-/-
elf3+/-
elf3+/+
Expected C1 C2 C3 C4 C5
n = 57 88 221 60 259
* * ns * *
B
rdu102
>90 dpf
% Genotype
Expected All C1 C2 C3 C4 C5
n = 313 90 22 62 68 71
* * ns * * *
C
rdu103
60 dpf
0.0077
25 50 25
14 64 22
Expected
Observed
n = 103
D
rdu103
60 dpf (heterozygous incross)
0.6434
0.9291
0.7447
Standard Length (cm)
elf3+/+ elf3+/- elf3-/-
n = 23 66 14
E
rdu103
60 dpf (homozygous incross)
0.2080
Standard Length (cm)
elf3+/+ elf3-/-
n = 55 37

Figure S5. *elf3*$^{-/-}$ adults of both alleles exhibit premature death. (**A-B**) Observed *elf3* allele genotyping cohorts [C] for rdu103 (**A**) and rdu102 (**B**) adults (> 90 dpf) compared to expected Mendelian outcomes. The data represented in (**A**) provide the cohort breakdown of the compiled genotyping presented in Fig.5A. (**C**) Comparison of observed genotypic ratios to expected Mendelian ratios of *elf3* genotypes (rdu103) at 60 dpf. These data represent the starting genotypic ratios for the longitudinal study in the heterozygous tanks referenced in Fig.5E (**D-E**) Standard length measurements of *elf3* genotypes in heterozygous (mixed genotype, **D**) or homozygous (*elf3*$^{+/+}$ or *elf3*$^{-/-}$ only, **E**) tanks at 60 dpf. These data represent the start of the longitudinal study referenced in Fig. 5F. *P*-values for (**A-B**) were calculated using a chi-square goodness-of-fit test and (**D&E**) using a one-way ANOVA with Tukey's multiple comparisons post-test.

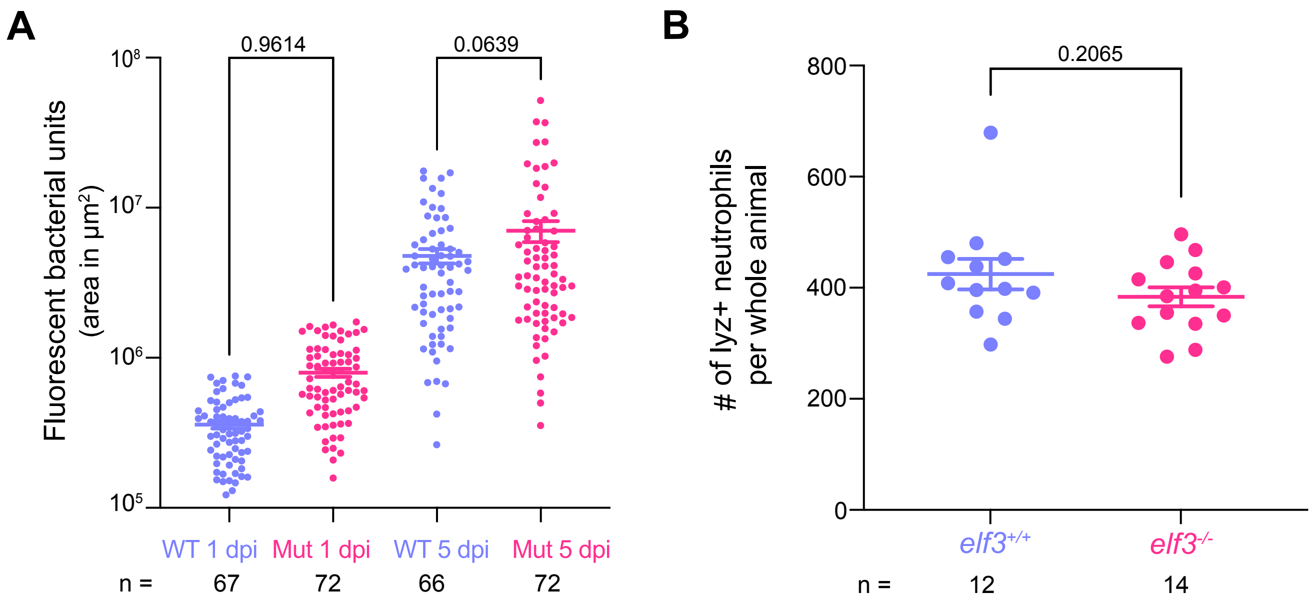
A
B
0.9614
0.0639
10^8
10^7
10^6
10^5
Fluorescent bacterial units
(area in µm²)
WT 1 dpi
Mut 1 dpi
WT 5 dpi
Mut 5 dpi
n =
67
72
66
72
0.2065
800
600
400
200
0
# of lyz+ neutrophils
per whole animal
elf3+/+
elf3-/-
n =
12
14

Figure S6. *elf3* mutant larvae have comparable numbers of neutrophils and exhibit similar burdens of *Mycobacterium marinum* following infection. (**A**) Comparison of the bacterial burden of fluorescent *Mycobacterium marinum* (*Mm:tdTomato*) in conventionally-reared *elf3* wild-type and mutant larvae 5 days after infection at 2dpf. 66-72 animals per condition. Representative of 2 experimental replicates. (**B**) Comparison of the number of lyz:EGFP$^+$ neutrophils in conventionally-reared *elf3* wild-type and mutant 6 dpf *Tg(lyz:EGFP)* larvae. *P*-values for (**A**) were calculated using a one-way ANOVA with Tukey's multiple comparisons post-test and (**B**) with an unpaired t-test.
